# Novel Dihydropyrimidinone Derivatives as Potential P-Glycoprotein Modulators

**DOI:** 10.1021/acsomega.1c05839

**Published:** 2022-05-02

**Authors:** Sabera Bijani, Faraz Shaikh, Sheefa Mirza, Shirley Weng In Siu, Nayan Jain, Rakesh Rawal, Nigel G. J. Richards, Anamik Shah, Ashish Radadiya

**Affiliations:** † Center of Excellence, National Facility for Drug Discovery Complex, Department of Chemistry, Saurashtra University, Rajkot 360005, India; ‡ Department of Chemistry, Marwadi University, Rajkot 360003, India; § Department of Computer and Information Science, University of Macau, Macau 999078, China; ∥ The Gujarat Cancer & Research Institute, Ahmedabad 380009, India; ⊥ Department of Internal Medicine, Faculty of Health Sciences, University of the Witwatersrand, Johannesburg 2193, South Africa; # Department of Life Sciences, School of Sciences, Gujarat University, Ahmedabad 380009, India; ∇ School of Chemistry, Cardiff University, Cardiff CF10 3AT, UK; ○ Astha, Saurashtra University Karmachari Cooperative Society, B/H Forensic Lab., Street No. 2, University Road, Rajkot 360005, India

## Abstract

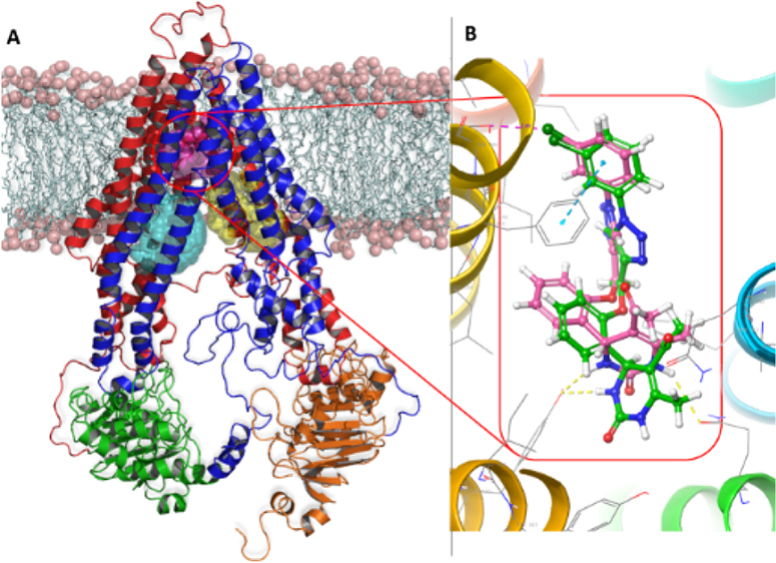

P-glycoprotein (Pgp), an ATP binding cassette (ABC) transporter, is an ATP-dependent efflux pump responsible for cancer multidrug resistance. As part of efforts to identify human Pgp (hPgp) inhibitors, we prepared a series of novel triazole-conjugated dihydropyrimidinones using a synthetic approach that is well suited for obtaining compound libraries. Several of these dihydropyrimidinone derivatives modulate human P-glycoprotein (hPgp) activity with low micromolar EC_50_ values. Molecular docking studies suggest that these compounds bind to the M-site of the transporter.

## 1 Introduction

Multidrug resistance (MDR) remains a deadly obstacle in anticancer treatments despite advances in understanding the molecular basis of this phenomenon. A key contributor to MDR is P-glycoprotein (Pgp), also known as multidrug resistance 1 protein (MDR1 gene), which is a membrane protein that couples ATP hydrolysis to the expulsion of small molecules, such as anticancer drugs, from the cell.^1^ The resulting decrease in intracellular drug concentration leads to reduced chemosensitivity and, ultimately, drug resistance. The ability of human P-glycoprotein (hPgp) to adopt multiple conformations and its substrate promiscuity, however, complicate efforts to design potent inhibitors using rational, structure-based strategies. This problem is reinforced by the fact that several binding sites are located within the TM helices, which are believed to form a pathway by which small molecules cross the membrane. To the best of our knowledge, no hPgp inhibitors have been approved for clinical use in the last four decades, including third generation candidates such as zosuquidar, elacridar, and tariquidar.^2^ New synthetic strategies are therefore needed to explore chemical space so as to obtain small molecules that can inhibit or at least modulate hPgp activity to overcome MDR.

In previous work in our laboratory, we found that functionalized 1,4-dihydropyridines had activity as hPgp inhibitors,^3^ which led to the discovery of compounds that combined anticancer activity with the ability to inhibit hPgp.^4^ Encouraged by this success, we are exploring whether alternate, synthetically available, and heterocyclic scaffolds represent novel leads for accessing hPgp inhibitors with improved potency. We now report that the dihydropyrimidinone (DHPM) scaffold can also be functionalized to give hPgp inhibitors by means of chemical transformations that can be easily adapted to prepare diverse DHPM libraries.

**Figure 1:**
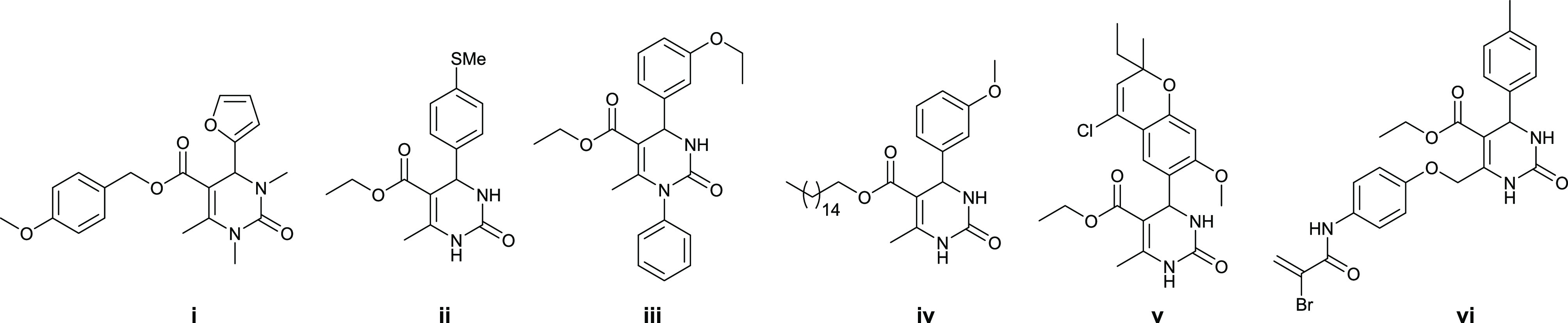
Structures of known DHPMs [**I–VI**] with anticancer activity (see text for details).

Our work on the DHPM scaffold is motivated by literature showing that DHPM derivatives exhibit activity against a variety of anticancer targets. For example, DHPM derivative **I** (Figure 1) inhibits the sodium iodide symporter, which is a transmembrane glycoprotein associated with many types of cancer.^5^ Oxo-monastrol analogue **II** and its derivatives are cytotoxic,^6^ and *N*-phenyl DHPM derivatives of **III** inhibit Hsp90 at nanomolar concentrations.^7^ Lipophilic DHPM variants, such as **IV**, exhibit antiproliferative properties against two glioma cell lines,^8^ and derivatives of DHPM **V** are active against lung cancer cell lines at nanomolar concentrations.^9^ Finally, aryl-α-haloacrylamide-functionalized DHPMs, such as **VI**, show anticancer activity in the micromolar range by inhibiting tubulin polymerization.^10^

## 2 Results and Discussion

**Figure 2:**
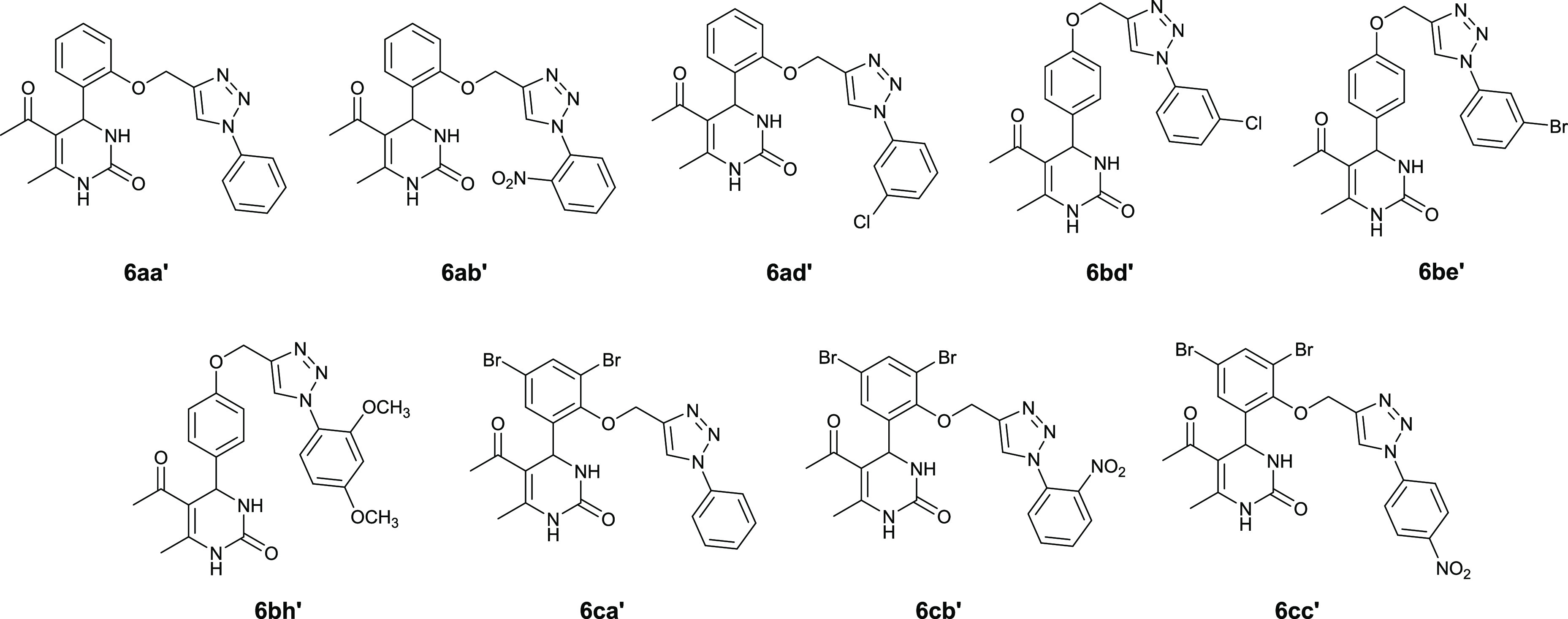
Structures of selected TRZ-DPHMs for which IC_50_ < 1.5 μM against the Caco-2 cell line. Structures and biological data for the complete set of TRZ-DPHMs are provided in the Supporting Information.

**Scheme 1:**
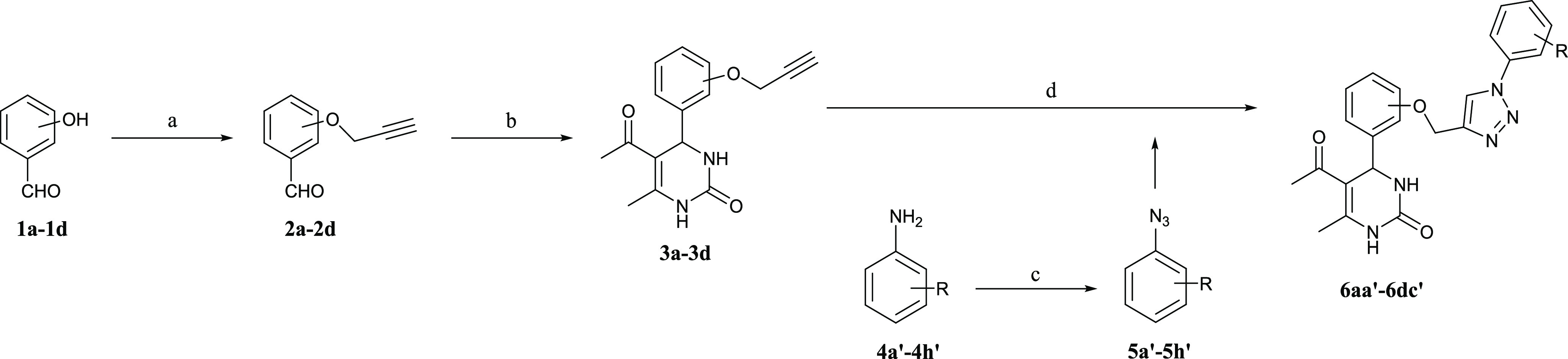
Synthetic Route for Preparing the Library of Novel DPHM Derivatives (a) Propargyl bromide and DMF; (b) acetylacetone, urea, and PEG-400; (c) HCl, NaNO_2_, and NaN_3_; (d) **5a′**–**5h′**, sodium ascorbate, CuSO_4_.5H_2_O, and (2:1:2) *t*-butanol:DMF:H_2_O.

Using a convergent synthetic sequence that combines the Biginelli reaction with CuAAC chemistry, we prepared a library of novel triazole-conjugated DHPM derivatives (TRZ-DHPMs) (Scheme 1). 1,2,3-Triazoles are easily prepared by copper-catalyzed alkyne–azide cycloaddition (CuAAC), thereby allowing the rapid construction of compounds capable of exhibiting a range of biological activities.^11,12^ As well as being straightforward to prepare, triazole substituents are present in clinically approved drugs^13^ and fungicides^14^ as well as in ligands that bind to a variety of cancer targets.^15,16^ Substituted hydroxybenzaldehydes **1a–1d** were propargylated in the presence of cesium carbonate to give benzaldehydes **2a**–**2d**,^17^ which were then reacted with acetyl acetone and urea following the Biginelli protocol^18^ to yield DHPM derivatives **3a**–**3d**. These alkynes were then connected to the substituted phenylazides **5a′**–**5h′** (prepared from the corresponding anilines **4a′**–**4h′**^19^) in the presence of CuSO_4_ and sodium ascorbate to give the target 4-aryl-5-acetyl-6-methyl-3,4-dihydropyrimidin-2(1*H*)-ones **6** as racemic mixtures (Figure 2 and Table S1, Supporting Information). This coupling reaction gave the best yields when a 2:1:2 *t*-butanol:DMF:water mixture was used as solvent (Table S2, Supporting Information).

With these compounds in hand, we characterized their cytotoxicity against a colorectal adenocarcinoma (Caco-2) cell line using an MTT-based assay. Half maximal inhibition concentration (IC_50_) values in the range of 0.6–3.7 μM were obtained for the library of functionalized TRZ-DHPMs (Table S3, Supporting Information), which compares favorably to those observed for known drugs such as carboplatin (7 μM), gemcitabine (5 μM) and daunorubicin (14 μM) under the same assay conditions (Table 1). The relatively small range of IC_50_ values observed for this series of functionalized TRZ-DHPMs complicates efforts to develop any SAR models but the presence of a halogen in the *N*-phenyl substitute of the triazole ring appears to correlate with sub-micromolar activity (e.g., **6ad′**, **6bd′**, and **6be′**). An *ortho*- rather than a *para*-relationship between the triazole and dihydropyrimidinone rings also appears important for increased cytotoxicity (Table S3, Supporting Information). The molecular basis for the cytotoxic activity of these TRZ-DHPMs, however, remains to be elucidated.

**Table 1:** Activities of Selected TRZ-DPHMs and Known Drugs

	compound	cytotoxicity,IC_50_ (μM)	inhibition of calceinextrusion, EC_50_ (μM)
1	**6aa′**	1.1 ± 0.2	12.56 ± 0.08
2	**6ab′**	1.3 ± 0.8	15.42 ± 0.06
3	**6ad′**	0.57 ± 0.03	**3.14 ± 0.03**
5	**6bd′**	0.76 ± 0.06	4.8 ± 0.2
6	**6be′**	0.8 ± 0.1	9.25 ± 0.02
7	**6bh′**	0.6 ± 0.2	4.2 ± 0.2
8	**6ca′**	0.9 ± 0.1	4.0 ± 0.2
9	**6cb′**	1.22 ± 0.08	3.4 ± 0.3
10	**6cc′**	1.25 ± 0.08	8.9 ± 0.1
11	carboplatin	7 ± 2	N.A.
12	gemcitabine	5 ± 1	N.A.
13	daunorubicin	14 ± 2	N.A.
14	verapamil	N.A.	9.52 ± 0.01
15	cyclosporin A	N.A.	5.36 ± 0.02

**Figure 3:**
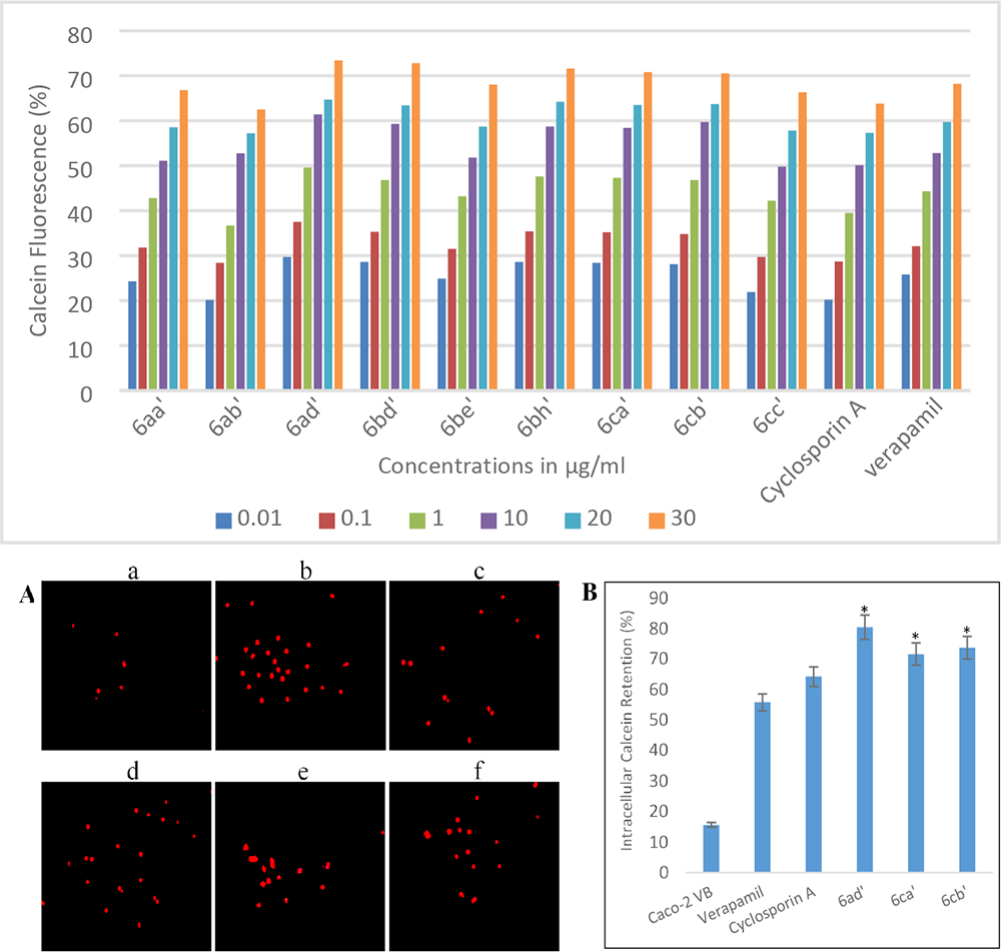
Top: Dose-dependent dye retention in Caco-2 VB cells treated with selected TRZ-DHPMs in the calcein-AM uptake assay. Bottom: (A) Fluorescence microscopy imaging of intracellular calcein in (a) untreated Caco-2 VB cells and Caco-2 VB cells 30 min after being treated with (b) TRZ-DHPM **6ad′**, (c) verapamil, (d) cyclosporin A, (e) TRZ-DHPM **6cb′**, and (f) TRZ-DHPM **6ca′**. TRZ-DHPM concentrations correspond to the relevant EC_50_ value (Table 1). (B) Graphical representation of calcein retention after treatment with TRZ-DHPMs **6ad′**, **6cb′**, and **6ca′**. Data are represented as mean ± SEM with *p* value (*p < 0.001) as compared to verapamil and cyclosporin A. Calcein retention in Caco-2 cells was used as a negative control. Full details are provided in the Supporting Information.

Despite a lack of information on the molecular mechanism of action, these nine cytotoxic TRZ-DHPMs were also assayed for their ability to reverse the hPgp-mediated efflux of calcein-AM, a lipophilic, non-fluorescent dye and a known Pgp substrate, from a Caco-2-VB cell line that expresses hPgp in much higher amounts and is resistant to vinblastine (Figure 3). In this assay, hydrolysis of intracellular calcein-AM to calcein by endogenous cellular esterases causes the cell to become highly fluorescent (see Figure S1 in Supporting Information). As a result, compounds that block the function of the hPgp efflux pump, which otherwise actively exports the dye from the cell, cause Caco-2-VB cells to accumulate calcein-AM, thereby increasing their fluorescence. The nine TRZ-DHPMs exhibiting the highest cytotoxicity were assayed for their ability to block calcein-AM release from Caco-2-VB cells (Table 1). The results of these experiments show that the TRZ-DHPMs **6ad′**, **6bd′**, **6be′**, **6bh′**, **6ca′**, **6cb′**, and **6cc′** exhibit similar or better efficacy than known Pgp substrates, cyclosporin A and verapamil (Table 1 and Figure 3). The most potent TRZ-DHPM, **6ad′**, has a chlorophenyl substituent attached to the triazole although replacing chlorine by an electron-withdrawing nitro group gives a 5-fold decrease in potency (**6ab′**). A similar decrease in potency is also observed when chlorine is replaced by hydrogen, raising the possibility that the ligand forms a σ-hole interaction with its target site in the ATP-dependent efflux pump. Remarkably, the presence of bromine substitution in the phenyl substituent of the dihydropyrimidi-none (**6cb′**) overcomes the effects of the nitro group and restores hPgp inhibition. Fluorescence microscopy measurements confirmed intracellular calcein retention when Caco-2-VB cells were treated with 1 μM calcein-AM in the presence of TRZ-DPHMs **6ad′**, **6ca′**, **6cb′**, cyclosporin A, and verapamil (Figure 3). Statistical analysis also showed that that **6ad′** (*p* < 0.0001) is a better modulator of hPgp function than either cyclosporin A or verapamil. Thus, these compounds have dual activity although their cytotoxicity is unlikely to be associated with their ability to modulate hPgp efflux activity.

**Figure 4:**
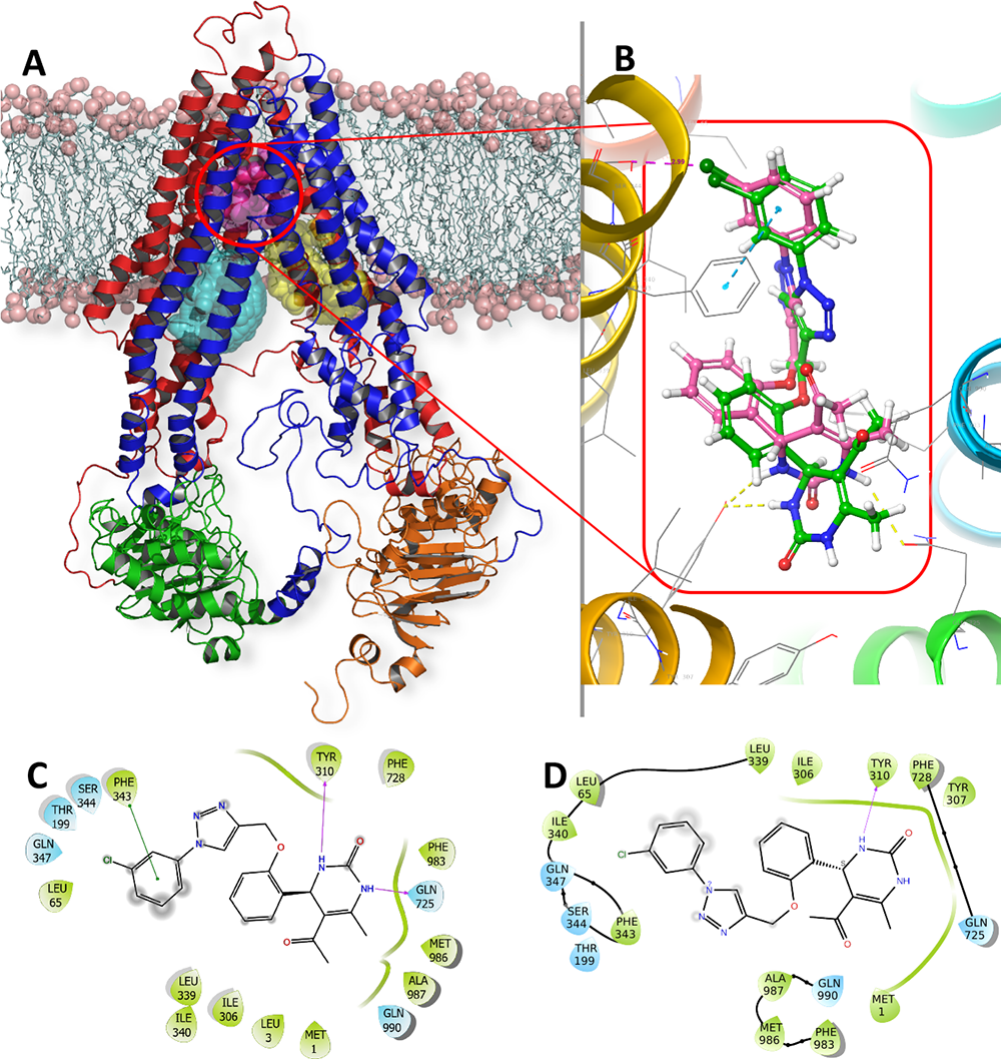
(A) Energy-minimized, inward-facing conformation of the hPgp homology model in a lipid bilayer, showing the homologous domains that exhibit pseudo-2-fold symmetry. The N-terminal transmembrane (TMD) and nucleotide binding (NBD) domains are colored red and green, respectively, and the C-terminal TMD and NBD are shown in blue and orange, respectively. M-, H-, and R-binding sites are rendered as magenta, cyan, and yellow spheres. Head and tail groups of the lipid bilayer membrane are represented by salmon spheres and pale cyan sticks, respectively. (B) Docking poses of the (*R*)- and (*S*)-enantiomers of **6ad′**. (C) Protein–ligand interactions computed for (*R*)-6ad′ bound at the M-site of hPgp. (D) Protein–ligand interactions computed for (*S*)-**6ad′** at the M-site of hPgp.

As in a recent study of small-molecule hPgp inhibitors,^20^ molecular modeling was used to obtain insights into how these DHPM derivatives might inhibit this transporter. Given the unavailability of high-resolution experimental coordinates for the “inward”-facing conformation of hPgp, we built a homology model of hPgp based on the experimental structure of *Mus musculus* Pgp, which has an 87% sequence match to hPgp,^21^ using well-precedented methods (see the Supporting Information).^22^ Energy minimization and equilibration using molecular dynamics (MD) simulations (100 ns) gave an inward-facing structure (Figure 4A) that is consistent with a low-resolution (3.6 Å) cryoEM structure of hPgp (occluded conformation).^23^ Molecular docking was used to examine how TRZ-DHPM **6ad′** might bind to the M-, R-, H-, and ATP-binding sites^24^ of the efflux pump. Thus, the (*R*) and (*S*) enantiomers of TRZ-DHPM **6ad′** were docked into the four potential binding sites of hPgp and evaluated using the scoring function implemented in GLIDE.^25^ We observed that (*S*)-**6ad′** exhibited little energetic preference for any of the binding sites (Table S4, Supporting Information), suggesting that this enantiomer might easily be transported out of the cell by hPgp. In contrast, the (*R*)- **6ad′** was predicted to bind more tightly to the M-site (−8.8 kcal mol^–1^) than to the H- and R-sites (−3.7 and – 6.5 mol^–1^). As neither enantiomer could be docked into the ATP-binding site, our docking calculations lead to the hypothesis that (*R*)-**6ad′** blocks hPgp activity, a hypothesis that will be tested in future experimental studies. In agreement with our hypothesis concerning σ-hole binding interactions (see above), we found that the best-scoring ligand pose placed the chloro substituent in (*R*)-**6ad′** within 3 Å of the side chain oxygen in Ser344 (Figure 4B).

Both enantiomers of **6ad′** exhibit π–π interactions with Phe343 and hydrogen-bond to Tyr310 when bound within the M-binding site of the hPgp model (Figure 4C,D). The origin of the stereochemical preference for M-site binding therefore appears to arise from the ability of (*R*)-**6ad′** to form an additional hydrogen bond to Gln725, a conserved residue in the access tunnel of the transporter (Figure 4C).^26^

## 3 Conclusions

These experimental and computational findings provide a firm basis for the development of functionalized DHPMs as hPgp inhibitors. Our synthetic route also provides a rapid entry into libraries that may yield small molecules with improved or altered selectivity for clinically relevant efflux pumps. Given the wide range of triazole substituents that be tolerated, our route can also introduce fluorophores for visualizing the binding of TRZ-DHPMs to hPgp and related transporters.^27^

## 4 Experimental Section

### 4.1 Materials

All the starting materials, reagents, and catalysts were purchased from Aldrich, Merck, or Lobachem and used without further purification. Anhydrous solvents (Spectrochem) were stored over molecular sieves. Chromatographic solvents used for the isolation/purification of compounds were distilled prior to use. Thin layer chromatography (TLC) was performed using 0.2 mm precoated plates of silica gel G60 F_254_ (Merck), and compounds were visualized with either with UV light (254 nm) or iodine vapor.

### 4.2 Apparatus

^1^H (400 MHz) and ^13^C (100 MHz) NMR spectra were recorded on a Bruker Avance III instrument using DMSO-*d*_6_, CDCl_3_, and MeOD-*d*_4_ as solvents. ^1^H and ^13^C chemical shifts are reported in ppm relative to tetramethylsilane (0.0). The following abbreviations designate chemical shift multiplicities: s = singlet, bs = broad singlet, d = doublet, dd = double doublet, t = triplet, dt = doublet of triplet, m = multiplet. All ^13^C NMR spectra are proton-decoupled. Melting points were determined in open capillary tubes and are uncorrected. Mass spectra were recorded on a Shimadzu GC–MS-QP-2010 Ultra spectrometer by direct injection. Elemental analyses were carried out on a Euro Vector EURO EA 3000 model.

### 4.3 Synthetic Procedures

#### 4.3.1 2-(Prop-2-ynyloxy)benzaldehyde (**2a**)^28^

2-Hydroxybenzaldehyde (10.0 g, 81.9 mmol, 1 equiv) was charged into a round-bottom flask containing DMF (30 mL) followed by the addition of Cs_2_CO_3_ (32.0 g, 98.2 mmol, 1.2 equiv), and the reaction was stirred at rt for 5–10 min. Propargyl bromide (8.0 mL, 90.09 mmol, 1.1 equiv) was added dropwise into the reaction mixture, and the reaction was stirred overnight at rt. After complete consumption of the starting material, the reaction mass was poured into crushed ice. The resulting precipitate obtained was collected by in vacuo filtration and washed with water. Light yellow solid; 11.5 g 88.0%; ^1^H NMR (DMSO-*d*_6_): δ 9.45 (s, 1H, CHO), 7.68–7.70 (m, 2H, ArH), 7.18–7.23 (m, 2H, ArH), 4.25 (s, 2H, CH_2_), 3.36 (s, 1H, CH).

The following compounds were prepared using the same synthetic procedure as described for **2a**.

#### 4.3.2 4-(Prop-2-yn-1-yloxy)benzaldehyde (**2b**)^28^

Compound **2b** was prepared from 4-hydroxybenzaldehyde (10.0 g, 81.9 mmol, 1 equiv) with Cs_2_CO_3_ (32.0 g, 98.2 mmol, 1.2 equiv) and propargyl bromide (8.0 mL, 90.09 mmol, 1.1 equiv). White solid; 11.7 g 89%; ^1^H NMR (DMSO-*d*_6_): δ 9.45 (s, 1H, CHO), 7.78 (d, *J* = 7.2 Hz, 2H, ArH), 7.23 (d, *J* = 7.2 Hz, 2H, ArH), 4.25 (s, 2H, CH_2_), 3.36 (s, 1H, CH).

#### 4.3.3 3,5-Dibromo-2-(prop-2-yn-1-yloxy)benzaldehyde (**2c**)^29^

Compound **2c** was prepared from 3,5-dibromo-2-hydroxy benzaldehyde (10.0 g, 35.9 mmol, 1 equiv), with Cs_2_CO_3_ (14.03 g, 43.0 mmol, 1.2 equiv) and propargyl bromide (3.5 mL, 39.4 mmol, 1.1 equiv). Yellow solid; 10.2 g, 90.2%; ^1^H NMR (DMSO-*d*_6_): δ 9.63 (s, 1H, CHO), 8.20 (s, 1H, ArH), 7.64 (s, 1H, ArH), 4.29 (s, 2H, CH_2_), 3.46 (s, 1H, CH).

#### 4.3.4 3-Methoxy-4-(prop-2-yn-1-yloxy)benzaldehyde (**2d**)^30^

Compound **2d** was prepared from 3-methoxy-4-hydroxy-benzaldehyde (10.0 g, 65.7 mmol, 1 equiv) with Cs_2_CO_3_ (25.6 g, 78.8 mmol, 1.2 equiv) and propargyl bromide (6.4 mL, 72.3 mmol, 1.1 equiv). Light yellow solid; 10.0 g, 80%; ^1^H NMR (DMSO-*d*_6_): δ 9.46 (s, 1H, CHO), 7.63 (s, 1H, ArH), 7.33–7.36 (m, 2H, ArH), 4.27 (d, 2H, CH_2_), 3.81 (s, 3H, CH_3_), 3.18 (t, *J* = 2.9, 1H CH).

#### 4.3.5 5-Acetyl-6-methyl-4-(2-(prop-2-yn-1-yloxy)phenyl)-3,4-dihydropyrimidin-2(1*H*)-one (**3a**)

Aldehyde **2a** (5.0 g, 32.0 mmol), urea (1.92 g, 32.0 mmol), acetyl acetone (3.2 mL, 32.0 mmol), and PEG (4.0 g) were mixed and heated at 100 °C for 3–5 h. After cooling to RT, the reaction mixture was poured into water, and the solid product was removed by filtration before being recrystalized from EtOH. White solid; 5.4 g, 59%; ^1^H NMR (DMSO-*d*_6_): δ 9.19 (s, 1H, NH), 8.96 (s, 1H, NH), 7.52 (m, 2H, ArH), 7.20 (m, 2H, ArH) 5.24 (s, 3H, 1 × CH_2_ and 1 × CH), 2.29 (s, 3H, COCH_3_), 2.09 (s, 3H, CH_3_). ^13^C NMR (DMSO-*d*_6_): δ 192.3, 155.2, 151.0, 147.3, 128.8, 127.3, 126.9, 125.6, 114.6, 108.5, 78.8, 75.8, 53.1, 51.3, 30.2, 18.8.

The following compounds were prepared using the same synthetic procedure as described for **3a**.

#### 4.3.6 5-Acetyl-6-methyl-4-(4-(prop-2-yn-1-yloxy)phenyl)-3,4-dihydropyrimidin-2(1*H*)-one (**3b**)

Compound **3b** was prepared from aldehyde **2b** (5.0 g, 32.0 mmol), urea (1.92 g, 32 mmol), acetyl acetone (3.2 mL, 32 mmol), and PEG (4.0 g). White solid; 4.5 g, 49.5%; ^1^H NMR (DMSO-*d*_6_): δ 9.19 (s, 1H, NH), 8.96 (s, 1H, NH), 7.52 (d, *J* = 7.8 Hz, 2H, ArH), 7.20 (d, *J* = 7.8 Hz, 2H, ArH) 5.24 (s, 3H, 1 × CH_2_ and 1 × CH), 2.29 (s, 3H, COCH_3_), 2.09 (s, 3H, CH_3_). ^13^C NMR (DMSO-*d*_6_): δ 192.3, 155.2, 151.0, 147.1, 136.8, 126.9, 114.6, 108.5, 78.8, 75.8, 53.1, 51.3, 30.2, 18.8.

#### 4.3.7 5-Acetyl-4-(3,5-dibromo-2-(prop-2-yn-1-yloxy)phenyl) -6-methyl-3,4-dihydropyrimidin-2(1*H*)-one (**3c**)

Compound **3c** was prepared from **2c** (6.5 g, 23.2 mmol), urea (1.39 g, 23.2 mmol), acetyl acetone (2.3 mL, 23.2 mmol), and PEG (4.0 g) Cream solid; 4.5 g, 44%; ^1^H NMR (DMSO-*d*_6_): δ 9.35 (s, 1H, NH), 9.05 (s, 1H, NH), 7.28 (s, 1H, ArH), 7.21 (s, 1H, ArH), 5.64 (s, 1H, CH), 5.44–5.31 (m, 1 × CH_2_ and 1 × CH), 2.37 (s, 3H, COCH_3_), 2.13 (s, 3H, CH_3_). ^13^C NMR (DMSO-*d*_6_): δ 193.1, 152.5, 150.4, 146.7, 131.6, 128.7, 125.5, 117.1, 115.5, 108.8, 77.8, 76.5, 55.3, 49.3, 27.6, 18.1.

#### 4.3.8 5-Acetyl-4-(2-methoxy-4-(prop-2-yn-1-yloxy)phenyl)-6-methyl-3,4-dihydropyrimidin-2(1*H*)-one (**3d**)

Compound **3d** was prepared from **2d** (4.0 g, 26.3 mmol), urea (1.58 g, 26.3 mmol), acetyl acetone (2.6 mL, 26.3 mmol), and PEG (4.0 g). Cream solid; 3.9 g, 47%; ^1^H NMR (DMSO-*d*_6_): δ 9.17 (s, 1H, NH), 8.92 (s, 1H, NH), 7.91 (d, *J* = 7.7 Hz, 2H, ArH), 7.79 (s, 1H, ArH), 5.22 (s, 1H, 1 × CH), 5.18 (s, 3H, 1 × CH_2_ and 1 × CH), 3.67 (s, 3H, OCH_3_), 2.20 (s, 3H, COCH_3_), 2.09 (s, 3H, CH_3_). ^13^C NMR (DMSO-*d*_6_): δ 194.4, 152.1, 148.0, 146.6, 143.8, 136.5, 128.7, 122.9, 120.1, 117.9, 110.9, 109.1, 61.6, 55.3, 53.4, 30.1, 18.8.

### 4.4 General Procedure for the Preparation of Phenylazides

A solution of aq. NaNO_2_ (5 mmol) was added dropwise to a solution of the aniline (5 mmol) dissolved in 6 N HCl (20 mL) so as to maintain the temperature within a range of 0–5 °C. After stirring at 0–5 °C for 1 h, a solution of aq. NaN_3_ (6 mmol, 1.2 equiv) was added dropwise to the reaction mixture. The reaction was then allowed to warm and stirred at RT for 6–8 h with progress being monitored by TLC. On completion, the reaction mixture was poured into cold water. The aqueous solution was extracted with EtOAc and the combined extracts were then washed with brine and aq. NaHCO_3_ before being dried over Na_2_SO_4_. Concentration of the solution under vacuum gave the desired phenylazide.

### 4.5 General Procedure for Synthesis of 5-Acetyl-6-methyl-Substituted 1,2,3-Triazol-4-yl)methoxy)phenyl)-3,4-dihydropyrimidin-2(1*H*)-one (**6aa′–6dc′**)

A mixture of substituted 5-acetyl-6-methyl-4-phenyl-3,4-dihydropyrimidin-2(1*H*)-one **3** (1 equiv) and phenylazide **5** (1.2 equiv) was charged into a sealed tube containing a solution of *tert*-butanol, DMF, and water (2:1:2, v/v, 5 mL). To this charged assembly, copper(II) sulfate (5 mol %) and sodium ascorbate (1 equiv) were added. The reaction was continued under stirring for 20–24 h at rt. After completion of the reaction (confirmed by TLC), the reaction mixture was filtered under vacuum and washed with water.

Using the above general synthetic procedure, compounds **6aa′–6dc′** were synthesized.

#### 4.5.1 5-Acetyl-6-methyl-4-(2-((1-phenyl-1H-1,2,3-triazol-4-yl)methoxy)phenyl)-3,4-dihydropyrimidin-2(1*H*)-one (**6aa′**)

Compound **6aa*′*** was prepared from **3a** (300 mg, 1.0 mmol) and azidobenzene (143 mg, 1.2 mmol). Pale yellow solid; 320 mg, 75%, Melting point: 145–147 °C. ^1^H NMR (DMSO-*d*_6_): δ 9.18 (s, 1H, NH), 8.96 (s, 1H, NH), 7.92 (d, *J* = 7.8 Hz, 2H, ArH), 7.64–7.60 (m, 2H, ArH) , 7.51 (d, *J* = 7.3 Hz, 1H, ArH), 7.35 (s, 1H, ArH), 7.30–7.27 , (m, 2H, ArH), 7.08 (d, *J* = 7.3 Hz, 1H, ArH), 6.95–6.92 (m, 1H, ArH), 5.66 (s, 1H, CH), 5.38 (s, 2H, CH_2_), 2.29 (s, 3H, COCH_3_), 2.00 (s, 3H, CH_3_). ^13^C NMR (DMSO-*d*_6_): δ 194.5, 154.8, 152.1, 148.3, 144.2, 136.5, 131.5, 129.9, 128.9, 128.7, 127.0, 122.5, 121.1, 120.1, 112.8, 107.7, 61.6, 48.6, 29.7, 18.6. MS (EI) *m/z* M^+^ 403.

#### 4.5.2 5-Acetyl-6-methyl-4-(2-((1-(2-nitrophenyl)-1H-1,2,3-triazol-4-yl)methoxy)phenyl)-3,4-dihydropyrimidin-2(1*H*)-one (**6ab′**)

Compound **6ab′** was prepared from **3a** (300 mg, 1.0 mmol) and **5b′** (197 mg, 1.2 mmol). Orange solid; 275 mg, 58%, Melting point: 133–135 °C. ^1^H NMR (DMSO-*d*_6_): δ 9.17 (s, 1H, NH), 8.89 (s, 1H, NH), 8.24 (d, *J* = 7.8 Hz, 1H, ArH), 8.00–7.96 (m, 1H, ArH), 7.93–7.91 (m, 1H, ArH) 7.86 (d, *J* = 7.4 Hz, 1H, ArH), 7.34, (s, 1H, ArH), 7.31–7.28 (m, 2H, ArH) , 7.10 (d, *J* = 7.3 Hz, 1H, ArH), 6. 97–6.94 (m, 1H, ArH) , 5.66 (s, 1H, CH), 5.38 (s, 2H, CH_2_), 2.29 (s, 3H, COCH_3_), 2.00 (s, 3H, CH_3_). ^13^C NMR (DMSO-*d*_6_): δ 194.5, 154.8, 152.1, 148.2, 144.1144.0, 134.3, 131.6, 131.2, 129.0, 128.9, 127.5, 127.1, 125.6, 125.5, 121.2, 112.9, 107.7, 61.6, 48.5, 29.7, 18.6. MS: *m/z* [M -C_9_H_7_N_4_O_2_]^+^ 245; Anal. calcd. for C_22_H_20_N_6_O_5_: C, 58.92; H, 4.50; N, 18.74. Found: C, 58.79; H, 4.61; N, 17.85 (%).

#### 4.5.3 5-Acetyl-6-methyl-4-(2-((1-(4-nitrophenyl)-1H-1,2,3-triazol-4-yl)methoxy)phenyl)-3,4-dihydropyrimidin -2(1*H*)-one (**6ac′**)

Compound **6 ac′** was prepared from **3a** (300 mg, 1.0 mmol) and **5c′** (197 mg, 1.2 mmol). Pale yellow solid; 332 mg, 70%, Melting point: 148–150 °C. ^1^H NMR (DMSO-*d*_6_): δ 9.17 (s, 2H, 2 × NH), 8.48 (d, *J* = 8.4 Hz, 2H, ArH), 8.25 (d, *J* = 8.4 Hz, 2H, ArH), 7.36–7.28 (m, 3H, ArH) , 7.09 (d, *J* = 7.1 Hz, 2H, ArH), 5.66 (s, 1H, CH), 5.39 (s, 2H, CH_2_), 2.29 (s, 3H, COCH_3_), 2.01 (s, 3H, CH_3_). ^13^C NMR (DMSO-*d*_6_): δ 194.4, 154.8, 152.1, 148.3, 146.7, 144.9, 140.7, 131.5, 128.9, 127.1, 125.5, 121.1, 120.6, 120.0, 112.8, 107.7, 61.5, 48.7, 29.7, 18.6. MS: *m/z* M^+^ 448; Anal. calcd. for C_22_H_20_N_6_O_5_: C, 58.92; H, 4.50; N, 18.74. Found: C, 58.73; H, 4.53; N, 17.67 (%).

#### 4.5.4 5-Acetyl-4-(2-((1-(3-chlorophenyl)-1H-1,2,3-triazol-4-yl)methoxy)phenyl)-6-methyl-3,4-dihydropyrimi-din-2(1*H*)-one (**6ad′**)

Compound **6ad′** was prepared from **3a** (300 mg, 1.0 mmol) and **5d′** (193 mg, 1.2 mmol). Pale cream solid; 340 mg, 73%, Melting point: 130–132 °C. ^1^H NMR (DMSO-*d*_6_): δ 9.20 (s, 1H, NH), 9.05 (s, 1H, NH), 8.06 (s, 1H, ArH), 7.96–7.94 (m, 1H, ArH) 7.64 (t, *J* = 8.0 Hz, 1H, ArH), 7.57 (d, *J* = 8.0 Hz, 1H, ArH), 7.75–7.26 (m, 3H, ArH) 7.12–7.10 (m, 1H, ArH) 7.11 (d, *J* = 7.4 Hz, 1H, ArH), 6.97–6.93 (m, 1H, ArH), 5.68 (s, 1H, CH), 5.38 (s, 2H, CH_2_), 2.31 (s, 3H, COCH_3_), 2.03 (s, 3H, CH_3_). ^13^C NMR (DMSO-*d*_6_): δ 194.4, 154.8, 152.1, 148.3, 144.4, 137.5, 134.1, 131.6, 131.5, 128.9, 129.5, 127.1, 122.7, 121.1, 119.8, 118.6, 112.8, 107.7, 61.6, 48.7, 29.7, 18.6. MS (EI) *m/z* [M - C_2_H_3_O]^+^ 394; Anal. calcd. for C_22_H_20_ClN_5_O_3_: C, 60.35; H, 4.60; N, 15.99. Found: C, 60.37; H, 4.56; N, 15.94 (%).

#### 4.5.5 5-Acetyl-6-methyl-4-(2-((1-(2,4,6-trichlorophenyl)-1H-1,2,3-triazol-4-yl)methoxy)phenyl)-3,4-dihydro-pyrimidin-2(1*H*)-one (**6af′**)

Compound **6af′** was prepared from **3a** (300 mg, 1.0 mmol) and **5f′** (267 mg, 1.2 mmol). Light yellow solid; 405 mg, 76%, Melting point: 139–141 °C. ^1^H NMR (DMSO-*d*_6_): δ 9.15 (s, 1H, NH), 8.78 (s, 1H, NH), 8.26–8.22 (m, 2H, ArH) , 7.30–7.24 (m, 3H, ArH), , 7.09–7.08 (m, 1H, ArH), 6.95 (s, 1H, ArH), 5.63 (s, 1H, CH), 5.38 (s, 2H, CH_2_), 2.28 (s, 3H, COCH_3_), 2.00 (s, 3H, CH_3_). ^13^C NMR (DMSO-*d*_6_): δ 194.4, 154.8, 152.0, 148.2, 143.4, 137.2, 134.0, 133.7, 131.7, 129.5, 128.9, 127.3, 126.6, 122.8, 121.1, 112.9, 107.7, 61.5, 48.6, 29.7, 18.6. MS: *m/z* M^+^ 505; Anal. calcd. for C_22_H_18_Cl_3_N_5_O_3_: C, 52.14; H, 3.58; N, 13.82. Found: C, 52.19; H, 3.56; N, 13.81 (%).

#### 4.5.6 5-Acetyl-6-methyl-4-(4-((1-phenyl-1H-1,2,3-triazol-4-yl)methoxy)phenyl)-3,4-dihydropyrimidin-2(1*H*)-one (**6ba′**)

Compound **6ba′** was prepared from **3b** (300 mg, 1.0 mmol) and **5a′** (143 mg, 1.2 mmol). Cream solid; 365 mg, 85%, Melting point: 133–135 °C. ^1^H NMR (DMSO-*d*_6_): δ 9.19 (s, 1H, NH), 8.96 (s, 1H, NH), 7.92 (d, *J* = 7.8 Hz, 2H, ArH), 7.80 (s, 1H, ArH), 7.62–7.58 (m, 2H, ArH) , 7.52–7.48 (m, 1H, ArH), 7.20 (d, *J* = 8.5 Hz, 2H, ArH), 7.04 (d, *J* = 8.5 Hz, 2H, ArH), 5.22 (s, 3H, 1 × CH_2_ and 1 × CH), 2.29 (s, 3H, COCH_3_), 2.09 (s, 3H, CH_3_). ^13^C NMR (DMSO-*d*_6_): δ 194.3, 157.2, 152.1, 147.8, 143.8, 136.8, 136.5, 129.8, 128.7, 127.6, 122.8, 120.1, 114.6, 109.5, 60.98, 53.2, 30.2, 18.8, MS: *m/z* M^+^ 403; Anal. calcd. for C_22_H_21_N_5_O_3_: C, 65.50; H, 5.25; N, 17.36. Found: C, 65.54; H, 5.27; N, 17.31 (%).

#### 4.5.7 5-Acetyl-6-methyl-4-(4-((1-(2-nitrophenyl)-1H-1,2,3-triazol-4-yl)methoxy)phenyl)-3,4-dihydropyrimidin-2(1*H*)-one (**6bb′**)

Compound **6bb′** was prepared from **3b** (300 mg, 1.0 mmol) and **5b′** (197 mg, 1.2 mmol). Orange solid; 345 mg, 73%, Melting point: 157–159 °C. ^1^H NMR (DMSO-*d*_6_): δ 9.20 (s, 1H, NH), 9.18 (s, 1H, NH), 8.75 (s, 1H, ArH), 8.43–8.33 (m, 2H, ArH) , 7.92–7.88 (m, 1H, ArH), , 7.80–7.78 (m, 1H, ArH), 7.19 (d, *J* = 7.9 Hz, 2H, ArH), 7.04 (d, *J* = 7.9 Hz, 2H, ArH), 5.24 (s, 2H, CH_2_), 5.22 (s, 1H, CH), 2.28 (s, 3H, COCH_3_), 2.09 (s, 3H, CH_3_). ^13^C NMR (DMSO-*d*_6_): δ 194.3, 157.1, 152.0, 148.4, 147.9, 144.3, 137.0, 136.8, 131.5, 127.6, 126.1, 123.2, 123.1, 120.3, 114.6, 109.5, 60.8, 53.1, 30.2, 18.8. MS: *m/z* M^+^ 448; Anal. calcd. for C_22_H_20_N_6_O_5_: C, 58.92; H, 4.50; N, 18.74. Found: C, 58.89; H, 4.53; N, 18.70 (%).

#### 4.5.8 5-Acetyl-6-methyl-4-(4-((1-(4-nitrophenyl)-1H-1,2,3-triazol-4-yl)methoxy)phenyl)-3,4-dihydropyrimidin-2(1*H*)-one (**6bc′**)

Compound **6bc′** was prepared from **3b** (300 mg, 1.0 mmol) and **5c′** (197 mg, 1.2 mmol). Orange solid; 351 mg, 74%, Melting point: 134–136 °C. ^1^H NMR (DMSO-*d_6_*) δ 9.18 (s, 2H, 2 × NH), 8.46 (d, *J* = 9.2 Hz, 2H, ArH), 8.25 (d, *J* = 9.2 Hz, 2H, ArH), 7.80 (s, 1H, ArH), 7.19 (d, *J* = 8.8 Hz, 2H, ArH), 7.04 (d, *J* = 8.8 Hz, 2H, ArH), 5.24 (s, 2H, CH_2_), 5.21 (s, 1H, CH), 2.28 (s, 3H, COCH_3_), 2.09 (s, 3H, CH_3_). ^13^C NMR(DMSO-*d_6_*) δ 194.3, 157.1, 152.0, 147.9, 146.7, 144.5, 140.7, 136.9, 127.6, 125.5, 123.2, 120.6, 114.6, 109.5, 60.8, 53.1, 30.2, 18.8. MS: *m/z* M^+^ 448; Anal. Calcd. for C_22_H_20_N_6_O_5_: C, 58.92; H, 4.50; N, 18.74. Found: C, 58.90; H, 4.51; N, 18.76 (%).

#### 4.5.9 5-Acetyl-4-(4-((1-(3-chlorophenyl)-1H-1,2,3-triazol-4-yl)methoxy)phenyl)-6-methyl-3,4-dihydropyrimidin-2(1*H*)-one (**6bd′**)

Compound **6bd′** was prepared from **3b** (300 mg, 1.0 mmol) and **5d′** (193 mg, 1.2 mmol). White solid; 380 mg, 82%, Melting point: 142–144 °C. ^1^H NMR (DMSO-*d*_6_): δ 9.22 (s, 1H, NH), 9.04 (s, 1H, NH), 8.07 (s, 1H, ArH), 7.93–7.95 (m, 1H, ArH), , 7.84 (s, 1H, ArH), 7.62–7.57 (m, 2H, ArH), 7.23 (d, *J* = 7.3 Hz, 2H, ArH), 7.06 (d, *J* = 7.3 Hz, 2H, ArH), 5.24 (s, 3H, 1 × CH_2_ and 1 × CH), 2.31 (s, 3H, COCH_3_), 2.11 (s, 3H, CH_3_). ^13^C NMR (DMSO-*d*_6_): δ 194.3, 157.2, 152.1, 147.9, 144.0, 137.5, 136.8, 134.1, 131.5, 128.4, 127.7, 122.9, 119.8, 118.6, 114.6, 109.5, 60.8, 53.2, 30.1, 18.8. MS: *m/z* M^+^ 437; Anal. calcd. for C_22_H_20_ClN_5_O_3_: C, 60.35; H, 4.60; N, 15.99. Found: C, 60.32; H, 4.58; N, 16.01 (%).

#### 4.5.10 5-Acetyl-4-(4-((1-(3-bromophenyl)-1H-1,2,3-triazol-4-yl)methoxy)phenyl)-6-methyl-3,4-dihydropyrimidin-2(1*H*)-one (**6be′**)

Compound **6be′** was prepared from **3b** (300 mg, 1.0 mmol) and **5e′** (251 mg, 1.2 mmol). Cream solid; 384 mg, 70%, Melting point: 138–140 °C. ^1^H NMR (DMSO-*d*_6_): δ 9.21 (s, 1H, NH), 9.04 (s, 1H, NH), 8.19 (s, 1H, ArH), 7.99–7.97 (m, 1H, ArH), 7.82 (s, 1H, ArH), 7.70 (d, *J* = 8.0 Hz, 1H, ArH), 7.56 (t, *J* = 8.0 Hz, 1H, ArH), 7.22 (d, *J* = 8.2 Hz, 2H, ArH), 7.05 (d, *J* = 8.2 Hz, 2H, ArH), 5.23 (s, 3H, 1 × CH_2_ and 1 × CH), 2.30 (s, 3H, COCH_3_), 2.10 (s, 3H, CH_3_). ^13^C NMR (DMSO-*d*_6_): δ 194.3, 157.1, 152.1, 147.9, 144.0, 137.6, 136.8, 131.7, 131.4, 127.7, 122.6, 119.0, 114.6, 109.5, 60.9, 53.2, 30.2, 18.8. MS: *m/z* [M -C_2_H_3_O]^+^ 438.

#### 4.5.11 5-Acetyl-6-methyl-4-(4-((1-(2,4,6-trichlorophenyl)-1H-1,2,3-triazol-4-yl)methoxy)phenyl)-3,4-dihydro-pyrimidin-2(1*H*)-one (**6bf′**)

Compound **6bf′** was prepared from **3b** (300 mg, 1.0 mmol) and **5f′** (267 mg, 1.2 mmol). Light yellow solid; 420 mg, 78%, Melting point: 135–137 °C. ^1^H NMR (DMSO-*d*_6_): δ 9.18 (s, 1H, NH), 8.73 (s, 1H, NH), 8.26 (s, 1H, ArH), 8.22 (s, 1H, ArH), 7.80 (s, 1H, ArH), 7.18 (d, *J* = 8.6 Hz, 2H, ArH), 7.03 (d, *J* = 8.6 Hz, 2H, ArH), 5.22 (s, 2H, CH_2_), 5.21 (s, 1H, CH), 2.28 (s, 3H, COCH_3_), 2.09 (s, 3H, CH_3_). ^13^C NMR (DMSO-*d*_6_): δ 194.3, 157.2, 152.0, 147.9, 142.9, 136.8, 134.0, 133.8, 131.6, 130.9, 129.7, 128.4, 127.6, 126.9, 114.6, 109.5, 60.8, 53.1, 30.2, 18.8. MS: *m/z* M^+^ 505; Anal. calcd. for C_22_H_18_Cl_3_N_5_O_3_: C, 52.14; H, 3.58; N, 13.82. Found: C, 52.11; H, 3.61; N, 13.80 (%).

#### 4.5.12 5-Acetyl-4-(4-((1-(2,4-dimethoxyphenyl)-1H-1,2,3-triazol-4-yl)methoxy)phenyl)-6-methyl-3,4-dihydro-pyrimidin-2(1*H*)-one (**6bh′**)

Compound **6bh′** was prepared from **3b** (300 mg, 1.0 mmol) and **5h′** (227 mg, 1.2 mmol). White solid; 400 mg, 82%, Melting point: 140–142 °C. ^1^H NMR (DMSO-*d*_6_): δ 9.18 (s, 1H, NH), 8.89 (s, 1H, NH), 7.80 (s, 1H, ArH), 7.48 (s, 1H, ArH), 7.43–7.41 (m, 1H, ArH), 7.19 (d, *J* = 8.0 Hz, 2H, ArH), 7.14–7.12 (m, 1H, ArH), 7.04 (d, *J* = 8.0 Hz, 2H, ArH), 5.20 (s, 3H, 1 × CH_2_ and 1 × CH), 3.86 (s, 3H, OCH_3_), 3.82 (s, 3H, OCH_3_), 2.29 (s, 3H, COCH_3_), 2.09 (s, 3H, CH_3_). ^13^C NMR (DMSO-*d*_6_): δ 194.2, 157.2, 152.1, 149.2, 148.8, 147.8, 143.5, 136.8, 129.9, 127.6, 122.8, 114.7, 114.6, 112.1, 109.5, 104.6, 61.0, 55.8, 55.7, 53.2, 30.2, 18.8. MS: *m/z* M^+^ 463.

#### 4.5.13 5-Acetyl-4-(3,5-dibromo-2-((1-phenyl-1H-1,2,3-triazol-4-yl)methoxy)phenyl)-6-methyl-3,4-dihydropyrimidin-2(1*H*)-one (**6ca′**)

Compound **6ca′** was prepared from **3c** (442 mg, 1.0 mmol) and **5a′** (143 mg, 1.2 mmol). Yellow solid; 263 mg, 47%, Melting point: 157–159 °C. ^1^H NMR (DMSO-*d*_6_): δ 9.35 (s, 1H, NH), 9.05 (s, 1H, NH), 7.97–7.95 (m, 2H, ArH), 7.86–7.85 (m, 1H, ArH), 7.74 (s, 1H, ArH), 7.65–7.61 (m, 2H, ArH), 7.54–7.52 (m, 1H, ArH), 7.28 (s, 1H, ArH), 5.74 (s, 1H, CH), 5.44–5.31 (m, 2H, CH_2_), 2.37 (s, 3H, COCH_3_), 2.18 (s, 3H, CH_3_).^13^C NMR (DMSO-*d*_6_): δ 194.1, 151.7, 151.4, 149.0, 143.7, 141.8, 136.5, 134.6, 129.8, 129.7, 128.7, 123.0, 120.2, 118.5, 117.7, 108.8, 66.4, 49.3, 30.6, 19.1. MS: *m/z* [M - C_9_H_8_N_3_]^+^ 403.

#### 4.5.14 5-Acetyl-4-(3,5-dibromo-2-((1-(2-nitrophenyl)-1H-1,2,3-triazol-4-yl)methoxy)phenyl)-6-methyl-3,4-dihydro-pyrimidin-2(1*H*)-one (**6cb′**)

Compound **6cb′** was prepared from **3c** (442 mg, 1.0 mmol) and **5b′** (197 mg, 1.2 mmol). Yellow solid; 273 mg, 45%, Melting point: 167–169 °C. ^1^H NMR (DMSO-*d*_6_): δ 9.34 (s, 1H, NH), 8.97(s, 1H, NH), 8.26–8.24 (m, 1H, ArH) , 8.01–7.81 (m, 4H, ArH), 7.75 (s, 1H, ArH), 7.28 (s, 1H, ArH), 5.74 (s, 1H, CH), 5.46–5.18 (m, 2H, CH_2_), 2.28 (s, 3H, COCH_3_), 2.18 (s, 3H, CH_3_). ^13^C NMR (DMSO-*d*_6_): δ 194.2, 151.5, 151.3, 149.0, 144.0, 143.3, 142.0, 134.5, 134.4, 131.2, 129.7, 129.0, 127.6, 126.2, 125.5, 118.5, 117.6, 108.9, 66.2, 49.3, 30.6, 19.1. MS: *m/z* [M - C_9_H_7_N_4_O_2_]^+^ 403.

#### 4.5.15 5-Acetyl-4-(3,5-dibromo-2-((1-(4-nitrophenyl)-1H-1,2,3-triazol-4-yl)methoxy)phenyl)-6-methyl-3,4-dihydro-pyrimidin-2(1*H*)-one (**6cc′**)

Compound **6cc′** was prepared from **3c** (442 mg, 1.0 mmol) and **5c′** (197 mg, 1.2 mmol). Brown solid; 273 mg, 45%, Melting point: 165–167 °C. ^1^H NMR (DMSO-*d*_6_): δ 9.36 (s, 1H, NH), 9.27 (s, 1H, NH), 8.48 (d, 2H, *J* = 8.2 Hz, ArH), 8.37 (d, 2H, *J* = 8.2 Hz, ArH), 7.85 (s, 1H, ArH), 7.74 (s, 1H, ArH), 7.28 (s, 1H, ArH), 5.73 (s, 1H, CH), 5.39 (dd, *J* = 12.0 Hz and 8.0 Hz, 2H, CH_2_), 2.38 (s, 3H, COCH_3_), 2.19 (s, 3H, CH_3_). ^13^C NMR (DMSO-*d*_6_): δ 194.1, 151.5, 149.1, 146.7, 144.3, 143.6, 141.8, 140.7, 134.6, 129.7, 125.3, 123.5, 120.5, 118.5, 117.6, 108.8, 66.2, 49.3, 30.6, 19.1.

#### 4.5.16 5-Acetyl-4-(3-methoxy-4-((1-phenyl-1H-1,2,3-triazol-4-yl)methoxy)phenyl)-6-methyl-3,4-dihydropyrimidin-2(1*H*)-one (**6da′**)

Compound **6da′** was prepared from **3d** (314 mg, 1.0 mmol) and **5a′** (143 mg, 1.2 mmol). White solid; 200 mg, 46%, Melting point: 151–153 °C. ^1^H NMR (DMSO-*d*_6_): δ 9.17 (s, 1H, NH), 8.92 (s, 1H, NH), 7.92–7.90 (m, 2H, ArH), 7.79 (s, 1H, ArH), 7.62–7.59 (m, 2H, ArH), 7.52–7.48 (m, 1H, ArH), 7.13–7.11 (m, 1H, ArH), 6.94 (s, 1H, ArH), 6.74–6.72 (m, 1H, ArH), 5.22 (s, 1H, CH), 5.18 (s, 2H, CH_2_), 3.67 (s, 3H, OCH_3_), 2.20 (s, 3H, COCH_3_), 2.09 (s, 3H, CH_3_). ^13^C NMR (DMSO-*d*_6_): δ 194.4, 152.1, 148.9, 148.0, 146.6, 143.8, 137.3, 136.5, 129.8, 128.7, 122.9, 120.1, 117.9, 113.7, 110.9, 109.1, 61.6, 55.3, 53.4, 30.1, 18.8. MS: *m/z* M^+^ 433; Anal. calcd. for C_23_H_23_N_5_O_4_: C, 63.73; H, 5.35; N, 16.16. Found: C, 63.71; H, 5.39; N, 16.14 (%).

#### 4.5.17 5-Acetyl-4-(3-methoxy-4-((1-(2-nitrophenyl)-1H-1,2,3-triazol-4-yl)methoxy)phenyl)-6-methyl-3,4-dihydropyri-midin-2(1*H*)-one (**6db′**)

Compound **6db′** was prepared from **3d** (314 mg, 1.0 mmol) and **5b′** (197 mg, 1.2 mmol). Yellow solid; 196 mg, 41%, Melting point: 153-155 °C. ^1^H NMR (DMSO-*d*_6_): δ 9.18 (s, 1H, NH), 8.83 (s, 1H, NH), 8.24–8.22 (m, 1H, ArH), 7.98–7.94 (m, 1H, ArH), 7.90–7.85 (m, 2H, ArH), 7.83–7.80 (m, 1H, ArH), 7.14–7.11 (m, 1H, ArH), 6.95 (s, 1H, ArH), 6.75–6.73 (m, 1H, ArH), 5.24 (s, 1H, CH), 5.20 (s, 2H, CH_2_), 3.73 (s, 3H, OCH_3_), 2.30 (s, 3H, COCH_3_), 2.10 (s, 3H, CH_3_). ^13^C NMR (DMSO-*d*_6_): δ 194.4, 152.1, 148.9, 148.0, 146.7, 144.0, 143.5, 137.4, 134.3, 131.1, 129.0, 127.6, 126.0, 125.5, 117.9, 113.7, 110.9, 109.1, 61.5, 55.3, 53.4, 30.1, 18.8. MS: m/z [M - C_2_H_3_O]^+^ 435; Anal. calcd. for C_23_H_22_N_6_O_6_: C, 57.74; H, 4.63; N, 17.56. Found: C, 57.78; H, 4.66; N, 17.51 (%).

#### 4.5.18 5-Acetyl-4-(3-methoxy-4-((1-(4-nitrophenyl)-1H-1,2,3-triazol-4-yl)methoxy)phenyl)-6-methyl-3,4-dihydro-pyrimidin-2(1H)-one (**6dc′**)

Compound **6dc′** was prepared from **3b** (314 mg, 1.0 mmol) and **5c′** (197 mg, 1.2 mmol). Brown solid; 210 mg, 44%, Melting point: 159–161 °C. ^1^H NMR (DMSO-*d*_6_): δ 9.18 (s, 1H, NH), 9.15 (s, 1H, NH), 8.46–8.44 (m, 2H, ArH), 8.25–8.24 (m, 2H, ArH), 7.79 (s, 1H, ArH), 7.13–7.11 (m, 1H, ArH), 6.95 (s, 1H, ArH), 6.74–6.72 (m, 1H, ArH), 5.22 (s, 3H, 1 × CH and 1 × CH_2_), 3.74 (s, 3H, OCH_3_), 2.30 (s, 3H, COCH_3_), 2.10 (s, 3H, CH_3_). ^13^C NMR (DMSO-*d*_6_): δ 194.4, 152.1, 148.9, 148.0, 146.7, 144.5, 140.7, 137.5, 125.5, 123.3, 120.6, 117.9, 113.7, 110.9, 109.1, 61.5, 55.3, 53.4, 31.2, 30.1, 18.8. MS: *m/z* M^+^ 478.

### 4.6 Biology

#### 4.6.1 Cell Culture

The colorectal adenocarcinoma Caco-2 cell line was generously provided by the Zydus Research Centre, Ahmedabad, Gujarat, India. These cells were maintained in DMEM (Hi-Media) containing 25 mM HEPES buffer, 1000 mg/L glucose, l-glutamine, sodium bicarbonate, and sodium pyruvate and supplemented with 20% heat-inactivated FBS (cell clone), 1% (v/v) minimal essential medium nonessential amino acids (Hi-Media), antibiotic, and antimycotic (penicillin, streptomycin, and amphotericin B; Hi-Media) in a humidified atmosphere of 5% CO_2_ at 37 °C. Cells were rinsed and split with trypsin–EDTA at 70–80% confluency, and only those cells exhibiting more than 90% viability were used in the biological assays. Caco-2 VB cells, which overexpress hPGP, were generated by growing Caco-2 cells in the presence of 1 μg/mL vinblastine. The drug was removed from the culture medium 2–3 days prior to using the Caco-2 VB cells in the biological assays.

#### 4.6.2 MTT Assay

Caco-2 cells were cultured in 96-well plates until they reached 70–80% confluency and then treated with the TRZ-DHPM derivatives (0.1–100 μM). Cytotoxicity was determined using an MTT (3-(4,5-dimethylthiazol-2-yl)-2,5-diphenyltetrazolium bromide) assay as described previously.^31^ Stock solutions of the TRZ-DHPM derivatives contained DMSO, but the final DMSO concentration in each assay did not exceed 0.1%.

#### 4.6.3 hPgp-Mediated Efflux Assay

hPgp-mediated efflux in Caco-2 VB cells was measured using the Vybrant Multidrug Resistance Assay Kit (V-13180, Thermo Fisher Scientific). Each well contained 100 μL of cells in culture medium, 50 μL of a solution containing the TRZ-DPHM derivative, and 50 μL of calcein-AM (total volume, 200 μL). The final concentration of putative hPGP inhibitor in each assay ranged from 0.01 to 30 μg/mL. Verapamil and cyclosporin A, both hPgp inhibitors, were used over the same concentration range as that used for the TRZ-DPHM derivatives. Caco-2 cells were used as a negative control (Figure S1). Cells were incubated at 37 °C for 15 min after the addition of TRZ-DPHM derivatives before 1 μM calcein-AM was added to each well (0.25 μM final concentration). After an additional 15 min at 37 °C, cells were washed with cold tissue culture medium (200 μL), and calcein retention was measured by the increase in intracellular fluorescence. PBS was used for all dilutions. All experiments were performed in triplicate.

#### 4.6.4 Cell Viability Assay

Cell viability was checked using a Trypan blue dye exclusion assay as described previously.^31^ In this assay, the % of viable cells was determined by Automated Cell Counter TC10 (BioRad) based on their capacity to uptake dye that differentiates live and dead cells. Live cells exclude dye due to their intact membrane, and dye penetrates the cell membrane of dead cells.

#### 4.6.5 Data Analysis

The effectiveness of inhibitors on Pgp efflux activity, i.e., calcein retention was calculated by the following equation
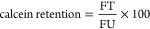
where FT is the fluorescence of treated cells and FU is the fluorescence of untreated cells.

### 4.7 Computational Methods

#### 4.7.1 Homology Modeling

A hPGp model was built using the multiple-template approach implemented in Modeller 9v14.^32^ Experimental coordinates 3G60 and 3G5U from *M. musculus* and 4F4C from *C. elegans* having high sequence similarity with human Pgp were used as templates for homology modeling.^33^ The preparation of the hPgp model involved hydrogen addition and the removal of water molecules, followed by minimization using the Impact module of Schrödinger. The quality of the hPGp model was assessed using the pdbsum server.^34^ A total of 96.4% residues were found to be in the allowed region with 86.7% in the most favored region.

#### 4.7.2 Molecular Dynamics Simulation

Molecular dynamics (MD) simulations were performed on the final hPgp model using the Desmond software package. Lipid bilayer position coordinates were obtained from the Orientations of Proteins in Membranes (OPM) database.^35^ The Desmond membrane insertion tab was then used to place the transmembrane (TM) coordinates. Further solvation of the model was done with TIP4P model in the cubic periodic boundary box. The resulting system of 76,326 atoms was minimized and pre-equilibrated by standard procedures implemented in Desmond. The distance between the box wall and hPgp model was set to be greater than 10 Å, thereby avoiding direct interactions with the periodic images. Steepest descent energy minimization was carried out until the gradient threshold (25 kcal/mol/Å), followed by L-BFGS (low-memory Broyden–Fletcher–Goldfarb–Shanno quasi-Newtonian minimizer) until a convergence threshold of 1 kcal/mol/Å was met. Further MD simulations were carried on the equilibrated systems for a desired period at a constant temperature of 300 K and constant pressure for the duration of 100 ns. The equations of motion were integrated with a 2 fs time step in the NPT ensemble. The SHAKE algorithm was applied to all hydrogen atoms, the van der Waals cutoff was 9 Å, and the temperature was maintained at 300 K, employing a Nose–Hoover thermostat with a relaxation time of 1 ps.

#### 4.7.3 Molecular Docking

The energy-minimized hPgp model was used in the docking study. R and S enantiomers of TRZ-DHPMs were docked to the three drug-binding sites categorized by Ferreira et al.^24^ as well as the ATP-binding site.^36^ Compound structures were prepared with the Ligprep module of Schrödinger. All three binding site grids were calculated using the Glide module of Schrödinger,^25^ and then the Glide XP docking was performed in all four binding sites individually.

## Supporting Information Available

Structures of all DHPMs prepared in this study; optimization of the CuAAC reaction and reaction conditions; analytical data of intermediates and other functionalized DHPMs prepared in this study; ^1^H, ^13^C, and mass spectra for selected functionalized DHPMs; fluorescence intensity of calcein in Caco-2 VB and Caco-2 cells; anticancer activities of all compounds; docking scores of selected DHPMs (PDF)

## ABBREVIATIONS

ATP: Adenosine triphosphate
ABC: ATP binding cassette
MDR: multidrug resistance
Pgp: P-glycoprotein
hPgp: human P-glycoprotein
MDR1: multidrug resistance 1
TM: transmembrane
DHPM: dihydropyrimidinone
CuAAC: copper-catalyzed alkyne–azide cycloaddition
DMF: dimethylformamide
PEG-400: polyethylene glycol 400
TRZ-DHPMs: triazole-conjugated DHPM derivatives
MTT: 3-(4,5-dimethylthiazol-2-yl)-2,5-diphenyl-2H-tetrazolium bromide
IC_50_: half maximal inhibition concentration
Caco-2 VB: vinblastine resistant Caco-2 cell lines that upregulate Pgp expression
SEM: standard error of the mean
TMD: transmembrane domain
NBD: nucleotide binding domain
MD: molecular dynamics
