# Supplementary material for: Novel Dihydropyrimidinone Derivatives as Potential P-Glycoprotein Modulators
Source: ACS Omega. 2022 May 2;7(19):16278–87. doi: 10.1021/acsomega.1c05839 (PMC9118204; doi:10.1021/acsomega.1c05839)
Supplement: Supplementary file 1 — ao1c05839_si_001.pdf [file ao1c05839_si_001.pdf]

Novel dihydropyrimidinone derivatives as potential P-glycoprotein modulators

Sabera Bijani,[‡,a,b] Faraz Shaikh,[‡,a,c] Sheefa Mirza,[d,e] Shirley Weng In Siu,[c] Nayan Jain,[f] Rakesh Rawal,[d,f] Nigel G. J. Richards,[g] Anamik Shah[a,h*] and Ashish Radadiya[a,g*]

a. Center of Excellence, National facility for Drug Discovery Complex, Department of Chemistry, Saurashtra University, Rajkot, India.
b. Department of Chemistry, Marwadi University, Rajkot.
c. Department of Computer and Information Science, University of Macau, Macau, China.
d. The Gujarat Cancer & Research Institute, Ahmedabad, India.
e. Department of Internal Medicine, Faculty of Health Sciences, University of the Witwatersrand, Johannesburg, South Africa.
f. Department of Life Sciences, School of Sciences, Gujarat University, Ahmedabad, Gujarat, India
g. School of Chemistry, Cardiff University, Cardiff, United Kingdom.
h. Astha, Street No. 2, Saurashtra University Karmachari Cooperative Society, B/H Forensic Lab., University Road, Rajkot, India

‡ Authors contributed equally.

Corresponding Authors:

Dr. Ashish Radadiya

National Facility of Drug Discovery,
Department of Chemistry,
Saurashtra University,
Rajkot, 360005
India

Present address:

School of Chemistry,
Cardiff University,
Cardiff, CF103AT
United Kingdom

Email: RadadiyaA@cardiff.ac.uk

Prof. Anamik Shah

National Facility of Drug Discovery &,
Department of Chemistry,
Saurashtra University,
Rajkot, 360005
India

Present address:

Astha, Street No. 2,
Saurashtra University Karmachari
Cooperative Society, B/H Forensic Lab.,
University Road, Rajkot, India

Email: anamik_shah@hotmail.com

SUPPORTING INFORMATION

Table of contents:

Table S1 – DHPM structures ……………...……………………………………………………….S3
Optimization conditions for the CuAAC reaction ……………………………………………...S5
Table S2 – Reaction Conditions …………………………………………………………….....S5
Spectroscopic Data for Bioactive DPHMs – ^{1}H & ^{13}C NMR and MS …….………………..........S6
Figure S1 – Fluorescence intensity of calcein in Caco-2 VB and Caco-2 cells ……….…..........S19
Table S3 – Anticancer activities of all DHPMs ……….. S20
Table S4 – Docking scores of all DHPMs ……………..…..S21

	Product		Product		Product
**6aa’**	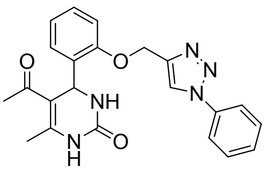<br>75%	**6ab’**	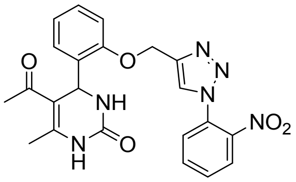<br>58%	**6ac’**	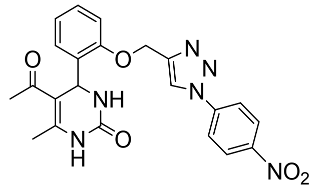<br>70%
**6ad’**	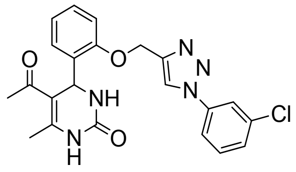<br>73%	**6ae’**	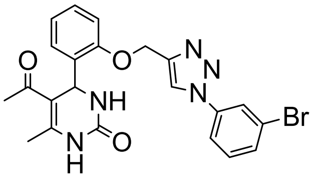<br>69%	**6af’**	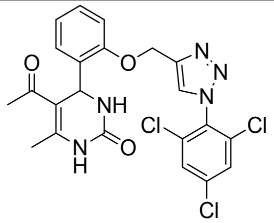<br>76%
**6ba’**	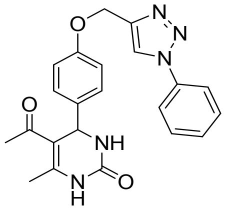<br>85%	**6bb’**	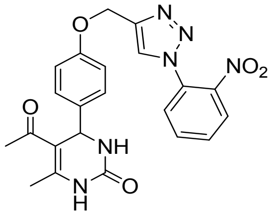<br>73%	**6bc’**	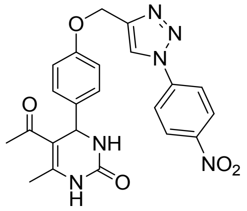<br>74%
**6bd’**	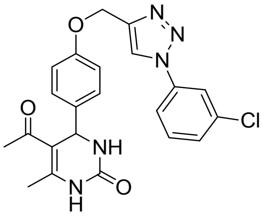<br>82%	**6be’**	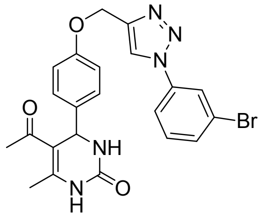<br>70%	**6bf’**	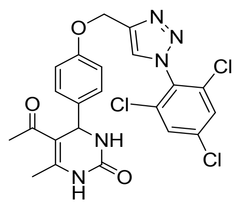<br>78%
**6bh’**	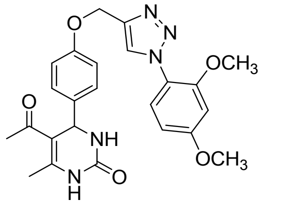<br>82%	**6ca’**	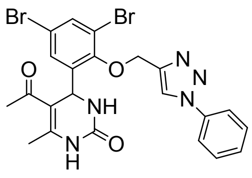<br>47%	**6cb’**	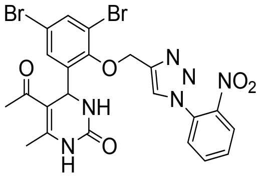<br>45%
**6cc’**	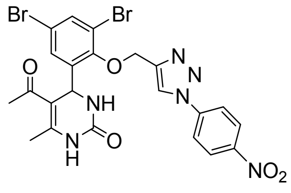<br>45%	**6da’**	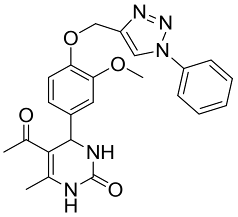<br>46%	**6db’**	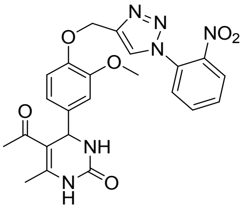<br>41%

**6dc'**	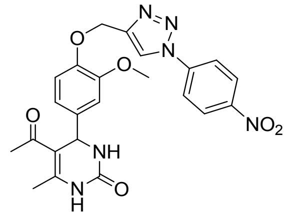<br>44%	

Table S1: Structures and overall yields of DHPMs prepared in this study

Optimization of conditions for the CuAAC reaction

Several organic solvents and their combinations were screened to optimize the yield of the Cu-catalysed coupling reaction between the DHPM **3a** and phenyl azide **5a′**. The reaction gave only trace amounts of the desired TRZ-DHPM when carried out in polar aprotic solvents, such as THF or acetonitrile (ACN) and 1,4-dioxane. Substantially increased yields were obtained, however, when the coupling was performed in MeOH or EtOH (Table 1) and these could be improved by the presence of water in the reaction mixture. After some experimentation, it was found that the combination of (2:1:2) tert-butanol, DMF, and water gave the highest yield of the disubstituted triazole (Table 1). This solvent combination was therefore used to prepare the set of TRZ-DHPMs in this study. The presence of water in the mixture likely solubilises $CuSO_4$ and ascorbate, thereby facilitating the reaction.

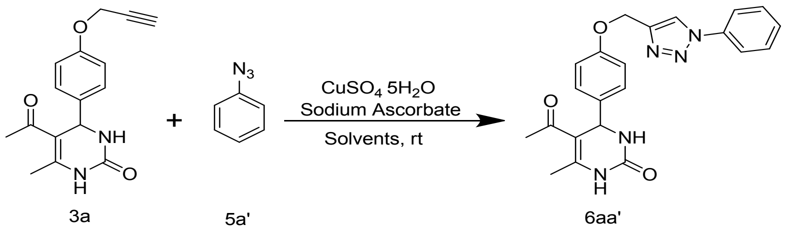

Entry	Solvents	Time (h)	% Yield[b]
1.	THF	48	Traces
2.	DMF	24	20
3.	ACN	36	Traces
4.	1,4-dioxane	48	Traces
5.	*tert*-butanol	36	55
6.	EtOH	24	42
7.	MeOH	24	45
8.	DMF + H_2O (1:1)	24	67
9.	EtOH+ H_2O (1:1)	24	58
10.	t-butanol + DMF + H_2O (1:1:1)	16	72
**11.**	**t-butanol + DMF + H_2O (2:1:2)**	**16**	**84**

Table S2: Reaction Conditions: [a]Starting materials **3a** (1 mmol), **5a′** (1.2 mmol), 0.2 eq of $CuSO_4$ $5H_2O$, and 0.1eq sodium ascorbate were reacted in the appropriate solvent in a sealed tube for the designated time mentioned in above table. [b]Isolated yields.

NMR Spectra

6aa′

^{1}H NMR

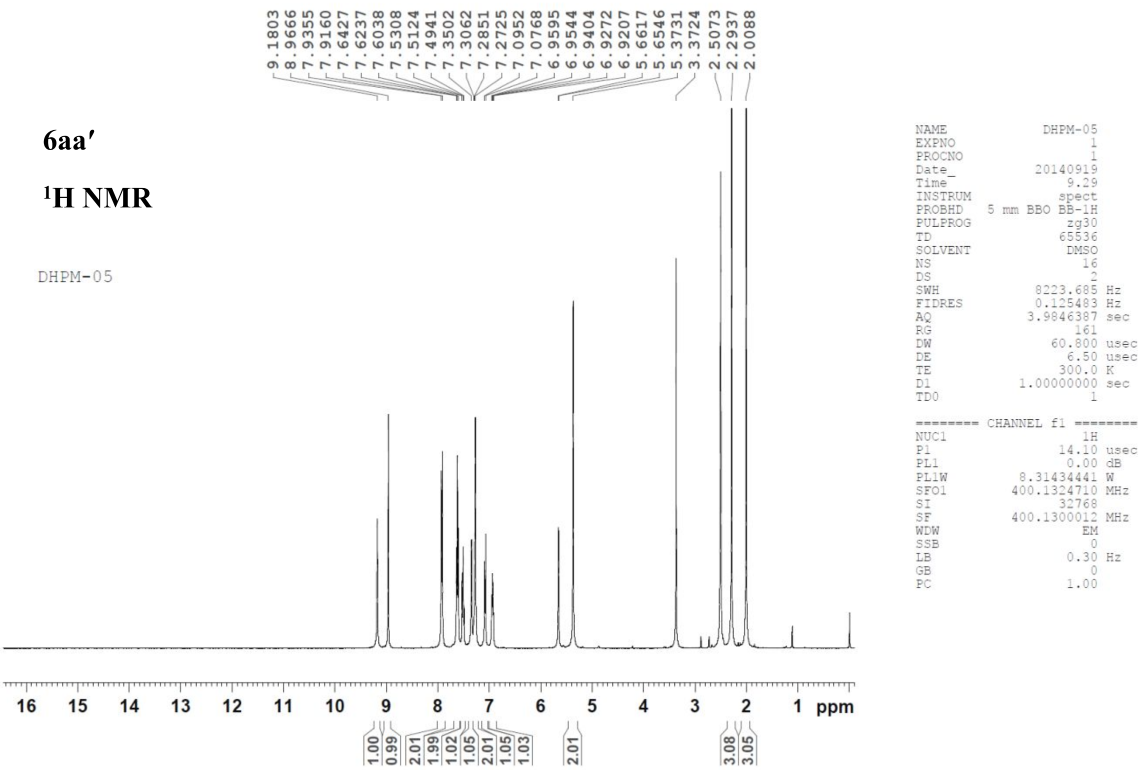

6aa′

^{13}C NMR

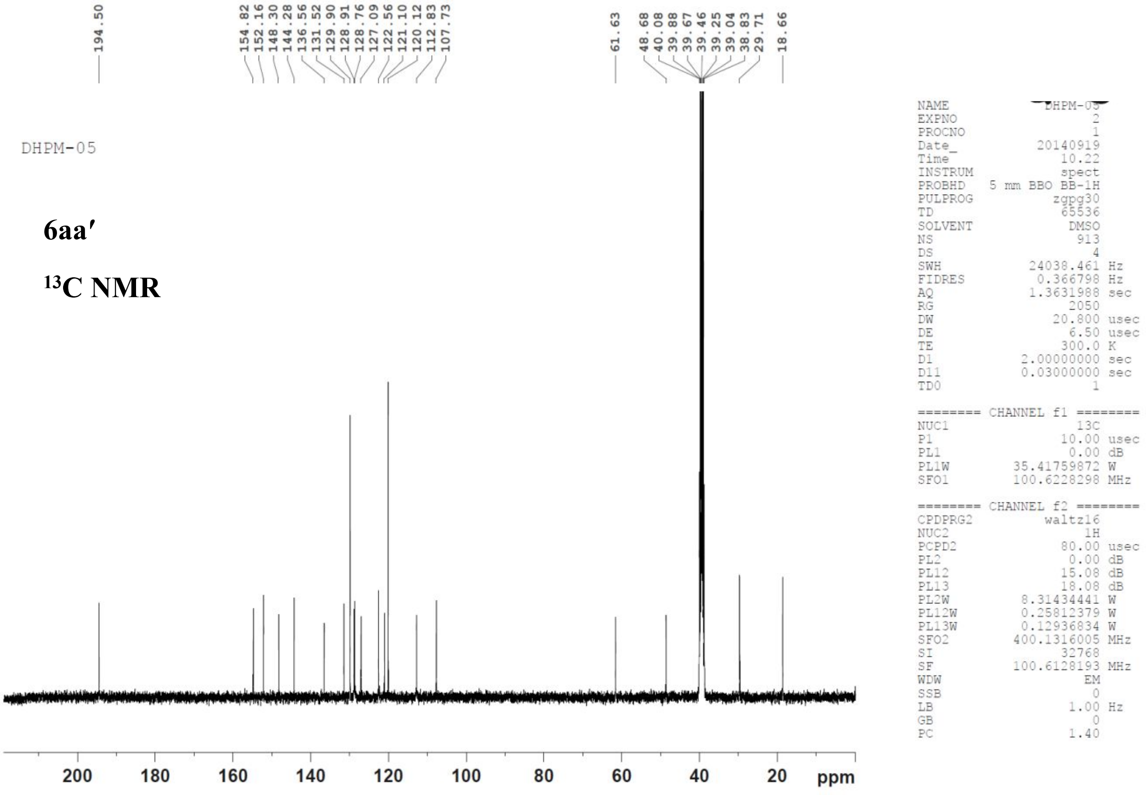

6ab′

^{1}H NMR

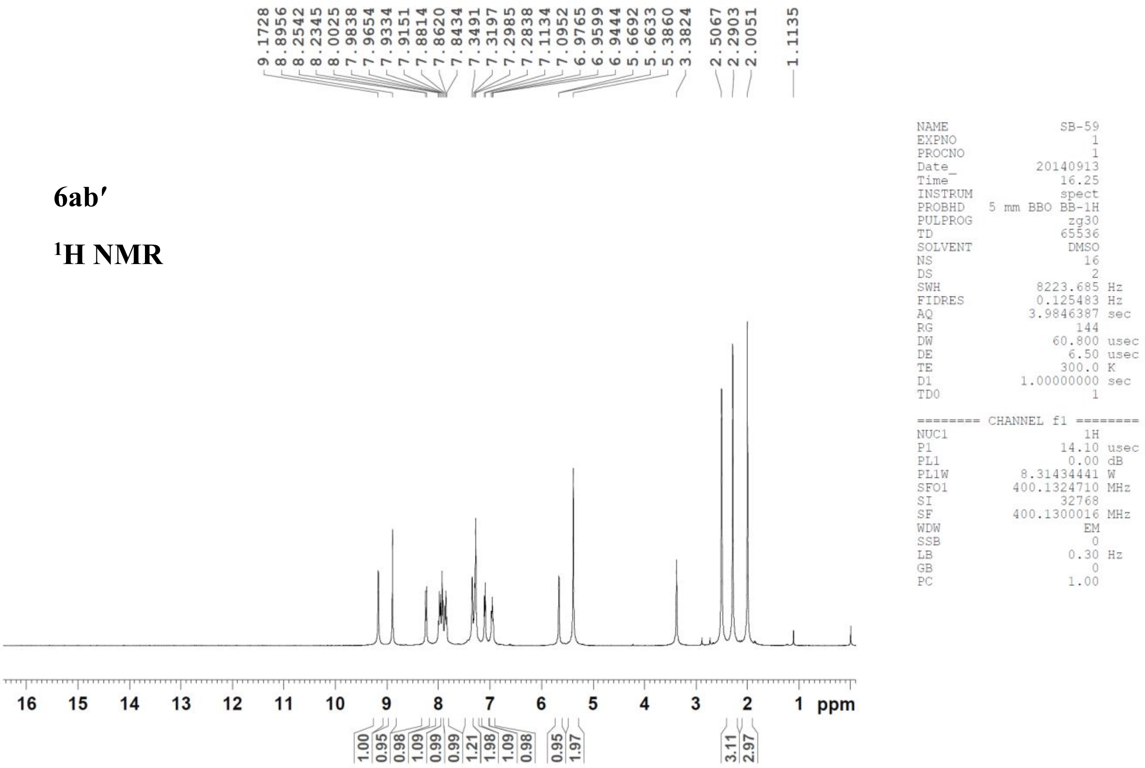

6ab′

^{13}C NMR

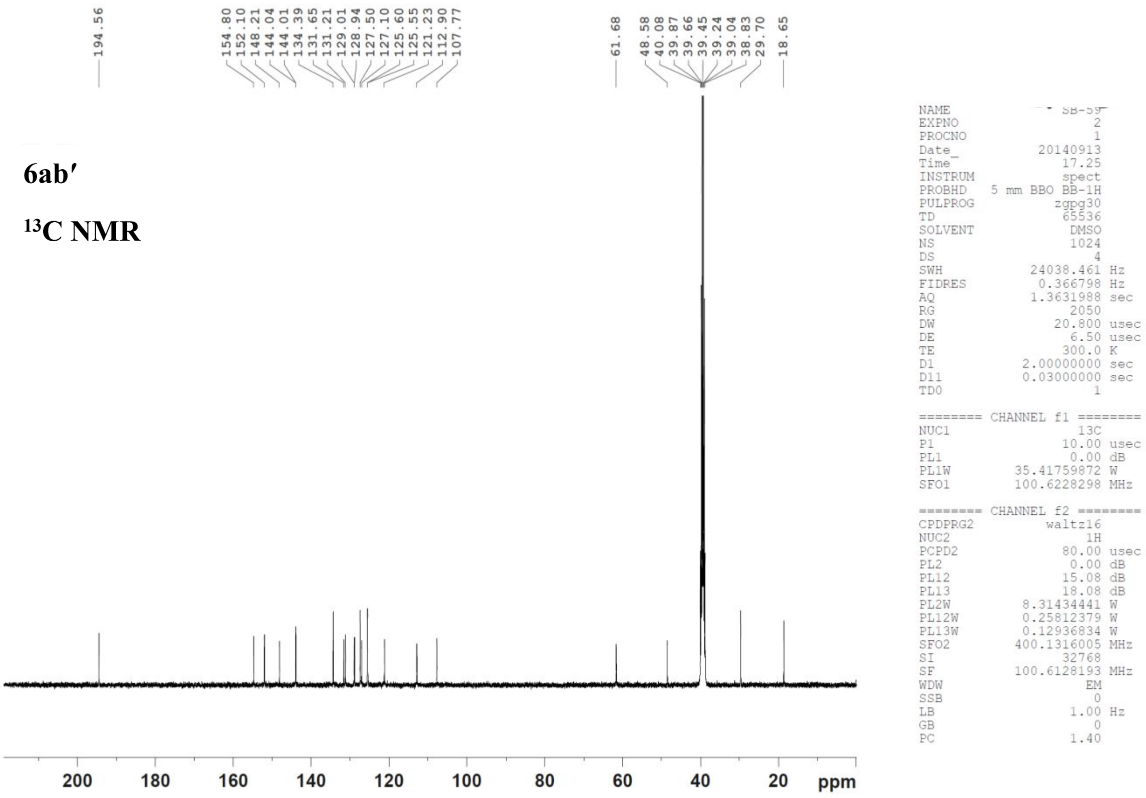

6ad′

^{1}H NMR

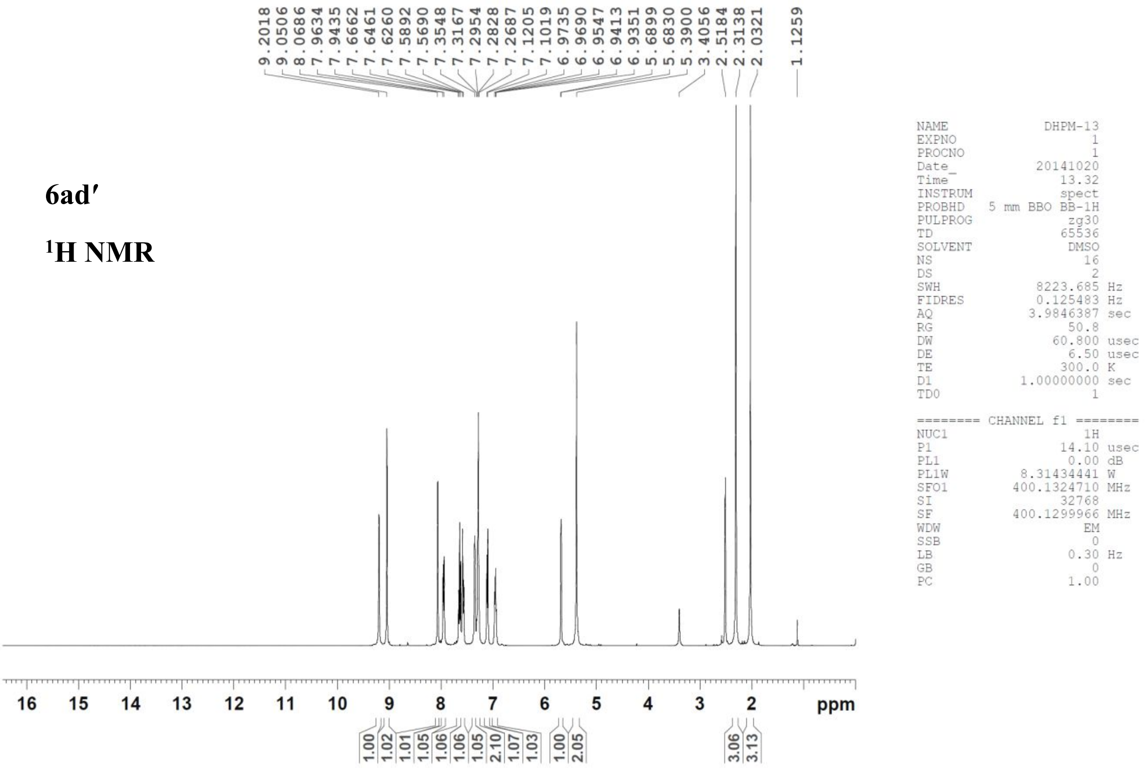

9.2018
9.0506
8.0686
7.9634
7.9435
7.6662
7.6461
7.6260
7.5892
7.5690
7.3548
7.3167
7.2954
7.2828
7.2687
7.1205
7.1019
6.9735
6.9690
6.9547
6.9413
6.9351
5.6899
5.6830
5.3900
3.4056
2.5184
2.3138
2.0321
1.1259
16 15 14 13 12 11 10 9 8 7 6 5 4 3 2 ppm
1.00 1.02 1.01 1.05 1.06 1.06 1.05 2.10 1.07 1.03 1.00 2.05 3.06 3.13
NAME DHPM-13
EXPNO 1
PROCNO 1
Date_ 20141020
Time 13.32
INSTRUM spect
PROBHD 5 mm BBO BB-1H
PULPROG zg30
TD 65536
SOLVENT DMSO
NS 16
DS 2
SWH 8223.685 Hz
FIDRES 0.125483 Hz
AQ 3.9846387 sec
RG 50.8
DW 60.800 usec
DE 6.50 usec
TE 300.0 K
D1 1.00000000 sec
TD0 1
======== CHANNEL f1 ========
NUC1 1H
P1 14.10 usec
PL1 0.00 dB
PL1W 8.31434441 W
SFO1 400.1324710 MHz
SI 32768
SF 400.1299966 MHz
WDW EM
SSB 0
LB 0.30 Hz
GB 0
PC 1.00

6ad′

^{13}C NMR

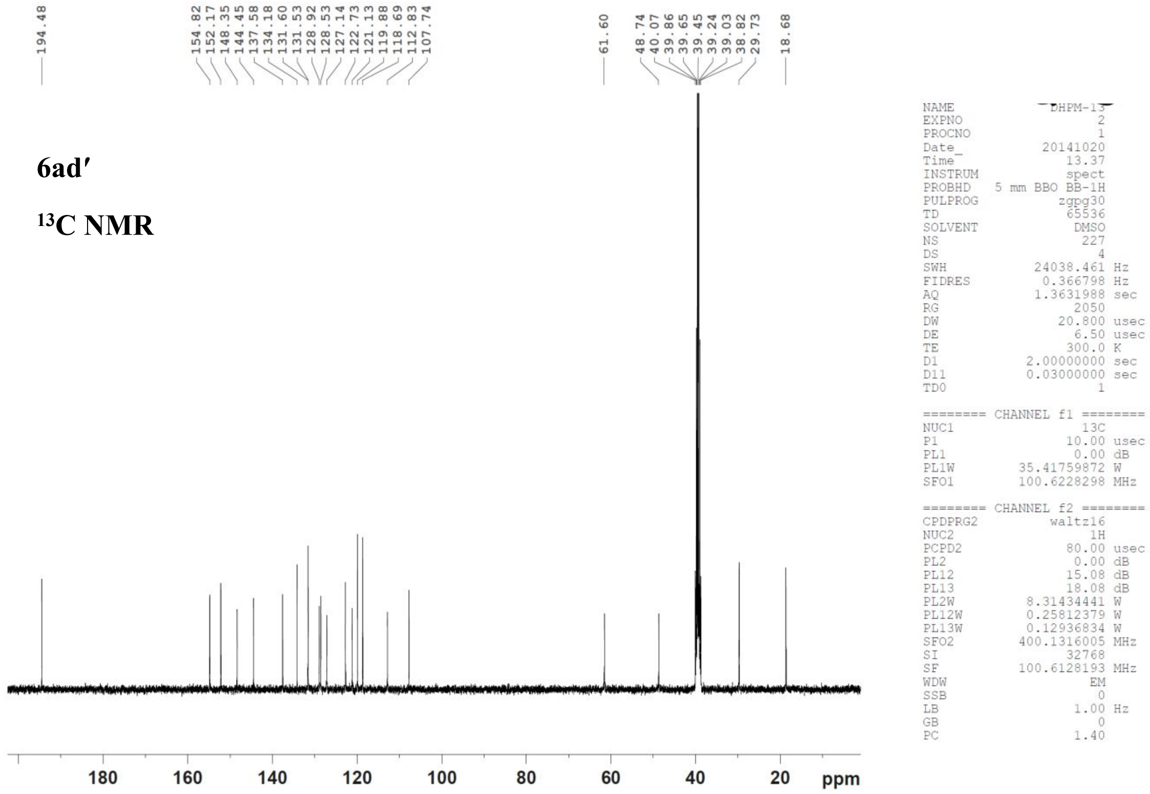

6bb′

^{1}H NMR

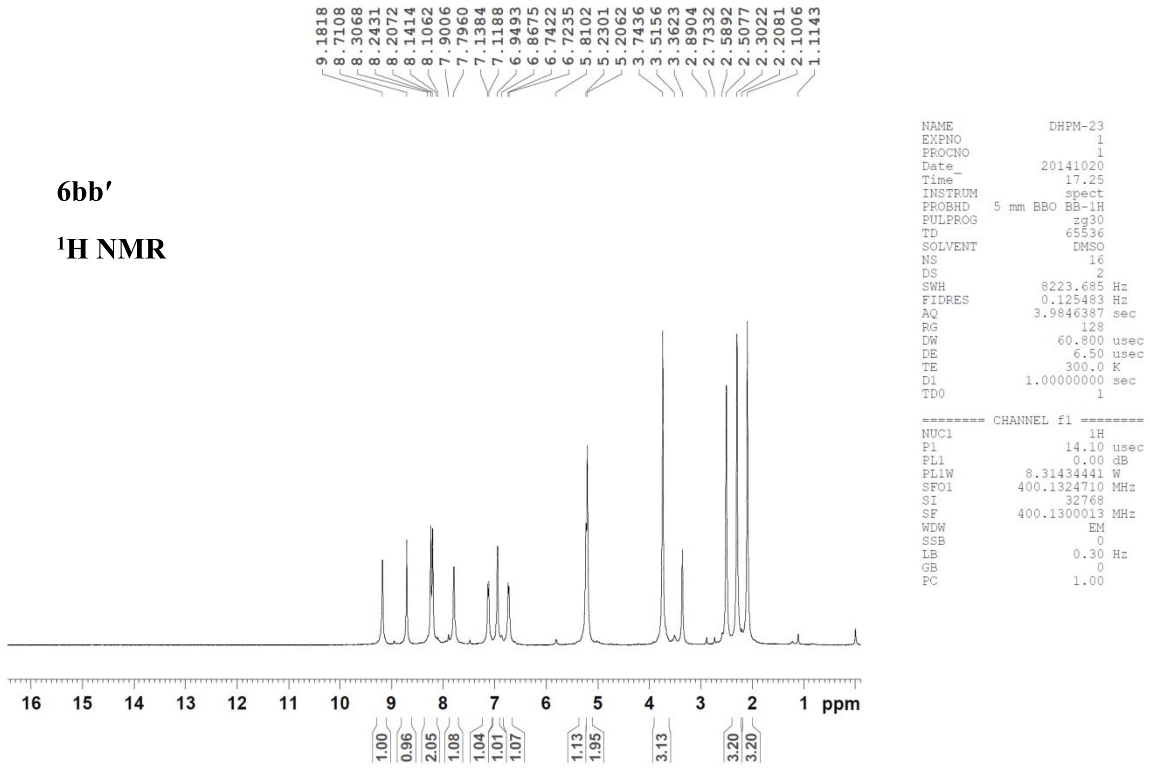

6bb′

^{13}C NMR

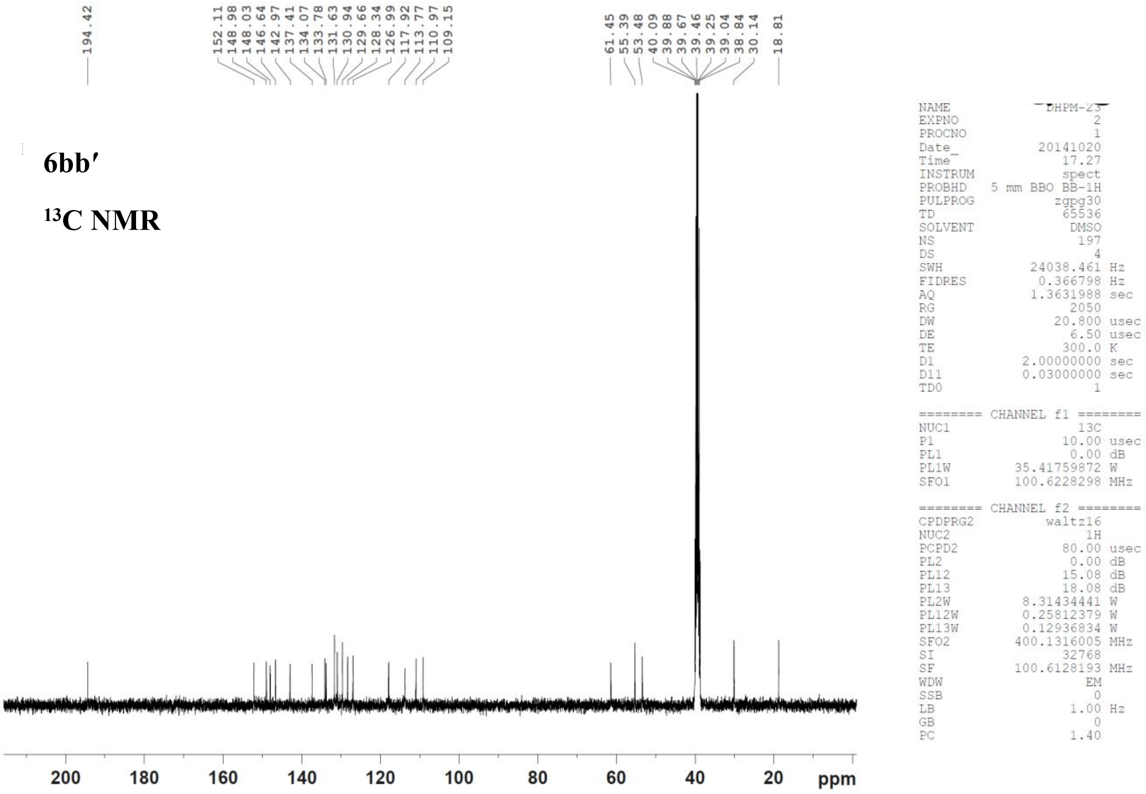

6bd′

^{1}H NMR

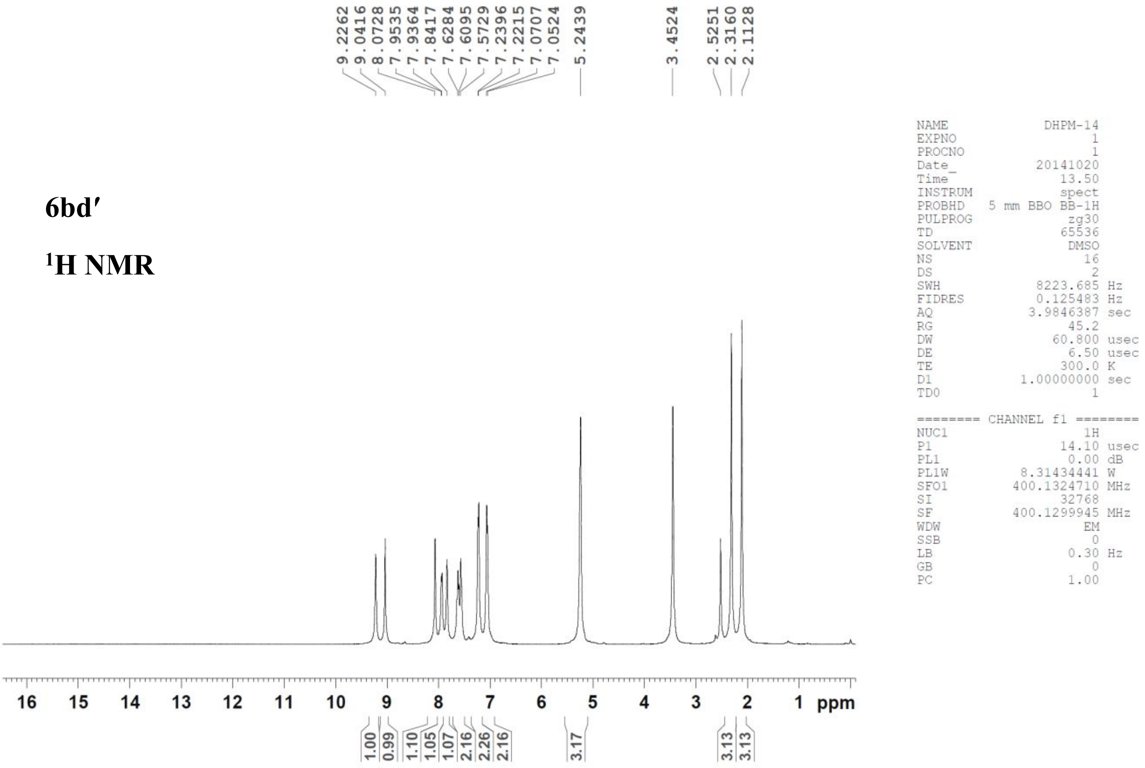

6bd′

^{13}C NMR

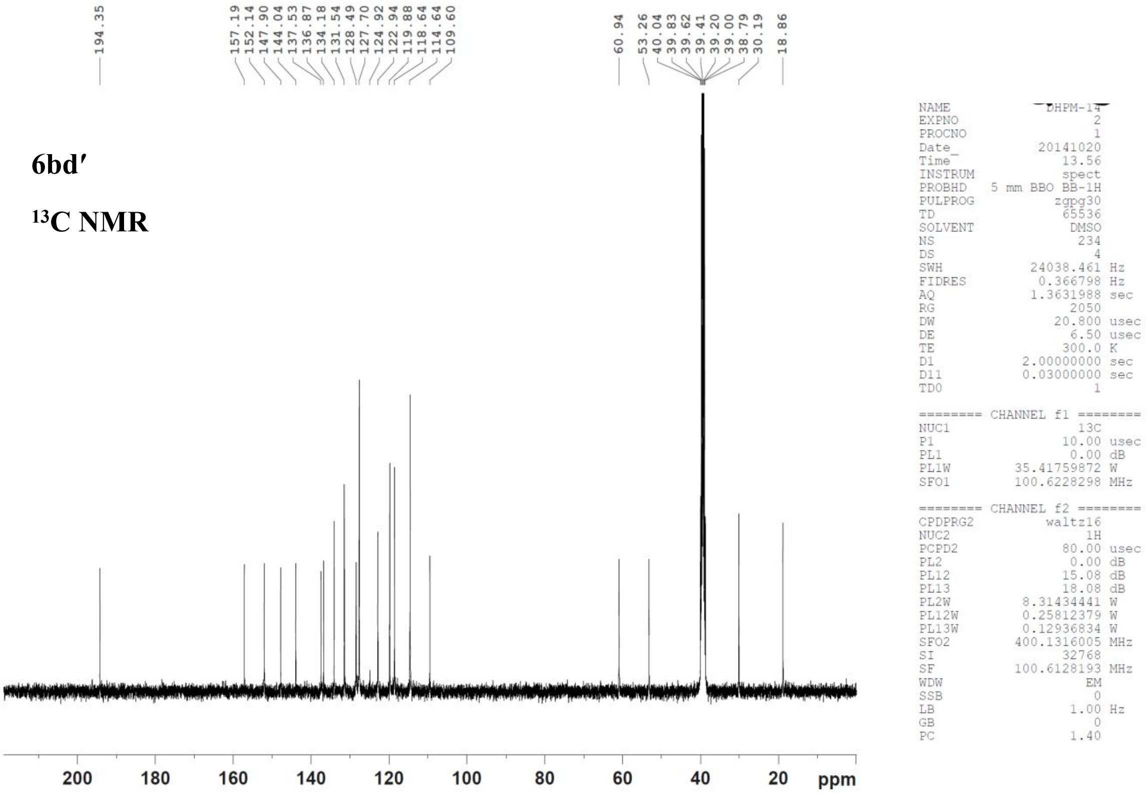
194.35
157.19
152.14
147.90
144.04
137.53
136.87
134.18
131.54
128.49
127.70
124.92
122.94
119.88
118.64
114.64
109.60
60.94
53.26
40.04
39.83
39.62
39.41
39.20
39.00
38.79
30.19
18.86
200
180
160
140
120
100
80
60
40
20
ppm
NAME DHPM-14
EXPNO 2
PROCNO 1
Date_ 20141020
Time 13.56
INSTRUM spect
PROBHD 5 mm BBO BB-1H
PULPROG zgpg30
TD 65536
SOLVENT DMSO
NS 234
DS 4
SWH 24038.461 Hz
FIDRES 0.366798 Hz
AQ 1.3631988 sec
RG 2050
DW 20.800 usec
DE 6.50 usec
TE 300.0 K
D1 2.00000000 sec
D11 0.03000000 sec
TD0 1
======== CHANNEL f1 ========
NUC1 13C
P1 10.00 usec
PL1 0.00 dB
PL1W 35.41759872 W
SFO1 100.6228298 MHz
======== CHANNEL f2 ========
CPDPRG2 waltz16
NUC2 1H
PCPD2 80.00 usec
PL2 0.00 dB
PL12 15.08 dB
PL13 18.08 dB
PL2W 8.31434441 W
PL12W 0.25812379 W
PL13W 0.12936834 W
SFO2 400.1316005 MHz
SI 32768
SF 100.6128193 MHz
WDW EM
SSB 0
LB 1.00 Hz
GB 0
PC 1.40

6be′

^{1}H NMR

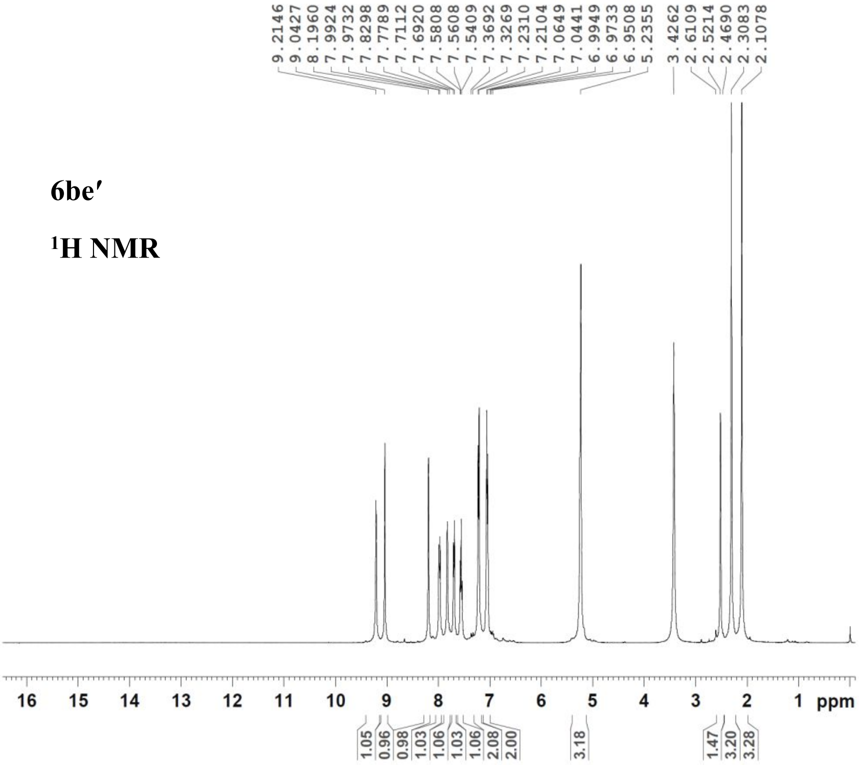


```
NAME            DHPM-15
EXPNO                 1
PROCNO                1
Date_          20141020
Time              14.09
INSTRUM           spect
PROBHD   5 mm BBO BB-1H
PULPROG            zg30
TD                65536
SOLVENT            DMSO
NS                   16
DS                    2
SWH            8223.685 Hz
FIDRES         0.125483 Hz
AQ            3.9846387 sec
RG                 50.8
DW               60.800 usec
DE                 6.50 usec
TE                300.0 K
D1           1.00000000 sec
TD0                   1

======== CHANNEL f1 ========
NUC1                 1H
P1                14.10 usec
PL1                0.00 dB
PL1W         8.31434441 W
SFO1        400.1324710 MHz
SI                32768
SF          400.1299956 MHz
WDW                  EM
SSB                   0
LB                 0.30 Hz
GB                    0
PC                 1.00
```

6be′

^{13}C NMR

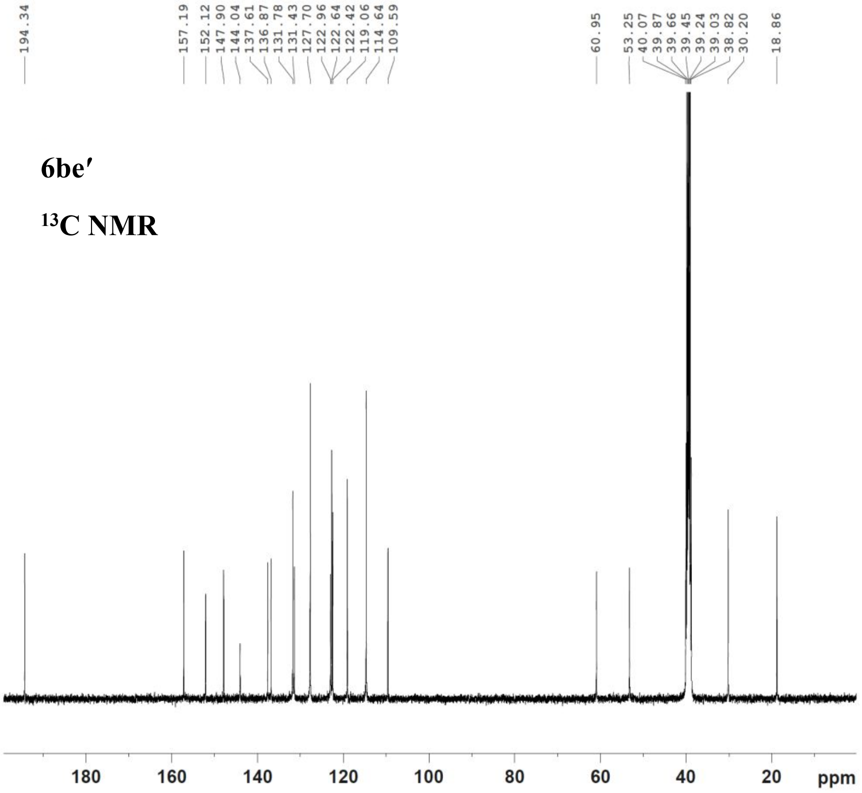


```
NAME            DHPM-15
EXPNO                 2
PROCNO                1
Date_          20141020
Time              14.57
INSTRUM           spect
PROBHD   5 mm BBO BB-1H
PULPROG          zgpg30
TD                65536
SOLVENT            DMSO
NS                  894
DS                    4
SWH           24038.461 Hz
FIDRES         0.366798 Hz
AQ            1.3631988 sec
RG                 2050
DW               20.800 usec
DE                 6.50 usec
TE                300.0 K
D1           2.00000000 sec
D11          0.03000000 sec
TD0                   1

======== CHANNEL f1 ========
NUC1                13C
P1                10.00 usec
PL1                0.00 dB
PL1W        35.41759872 W
SFO1        100.6228298 MHz

======== CHANNEL f2 ========
CPDPRG2         waltz16
NUC2                 1H
PCPD2             80.00 usec
PL2                0.00 dB
PL12              15.08 dB
PL13              18.08 dB
PL2W         8.31434441 W
PL12W        0.25812379 W
PL13W        0.12936834 W
SFO2        400.1316005 MHz
SI                32768
SF          100.6128193 MHz
WDW                  EM
SSB                   0
LB                 1.00 Hz
GB                    0
PC                 1.40
```

6da′

^{1}H NMR

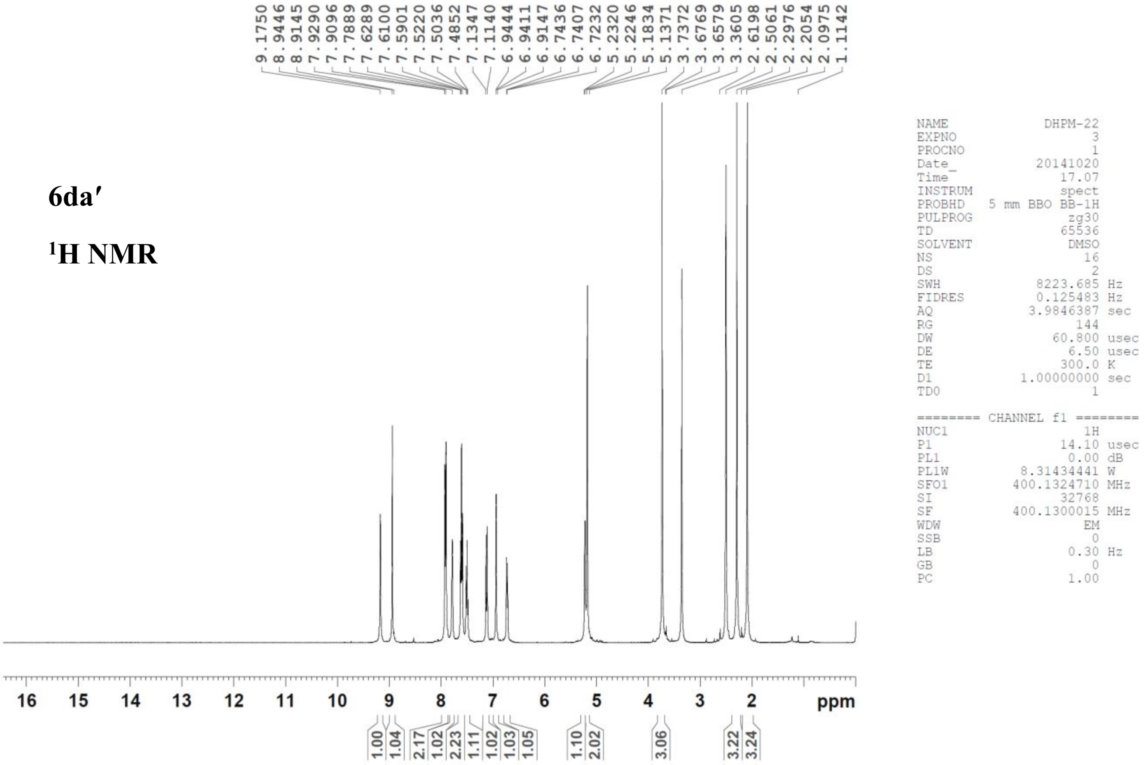

6da′

^{13}C NMR

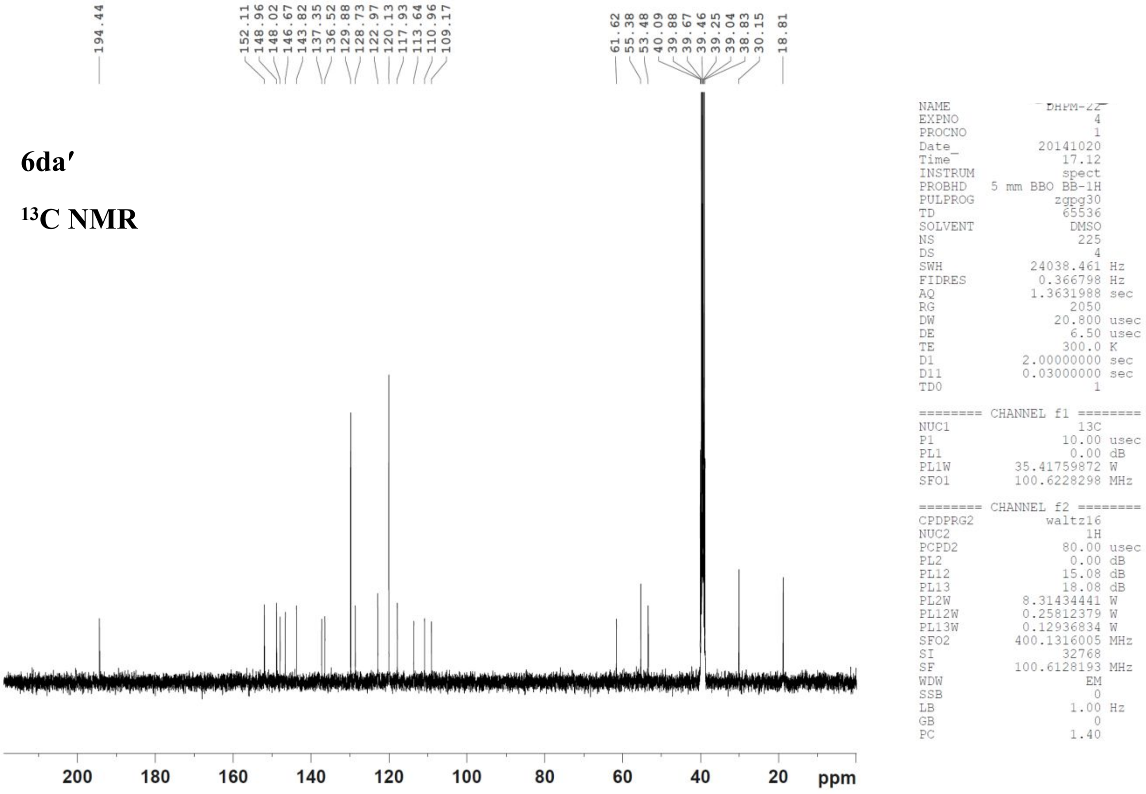

6db′

^{1}H NMR

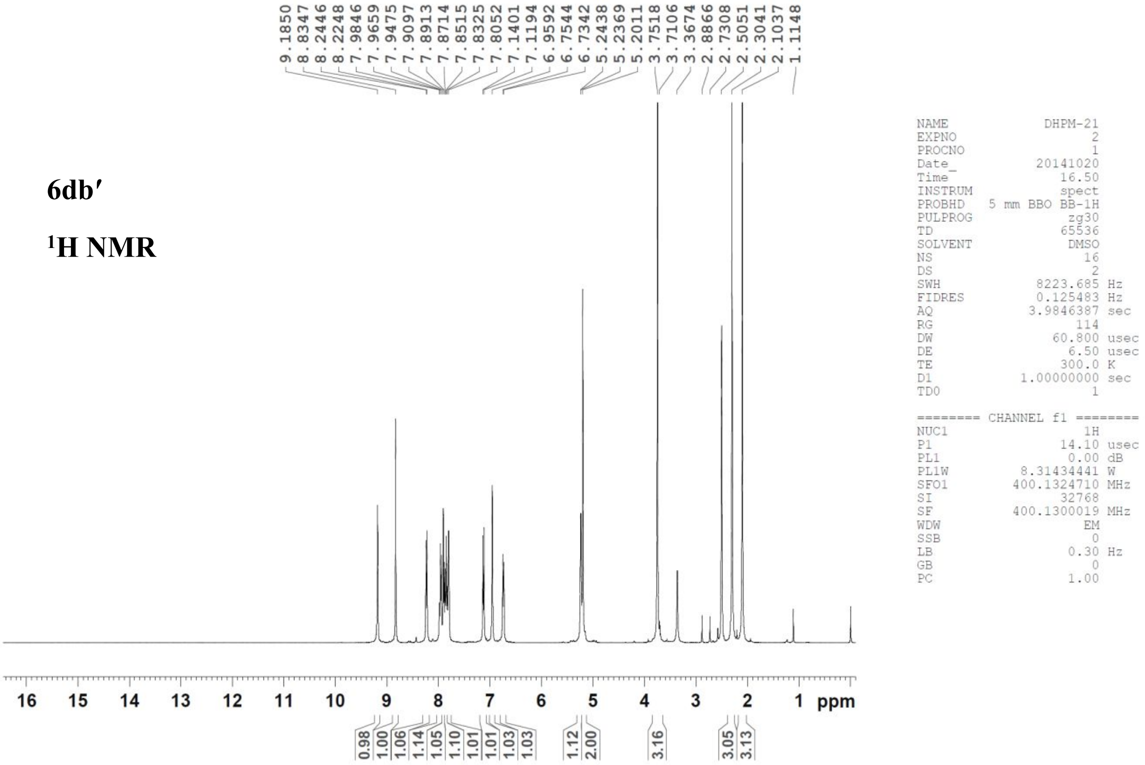

6db′

^{13}C NMR

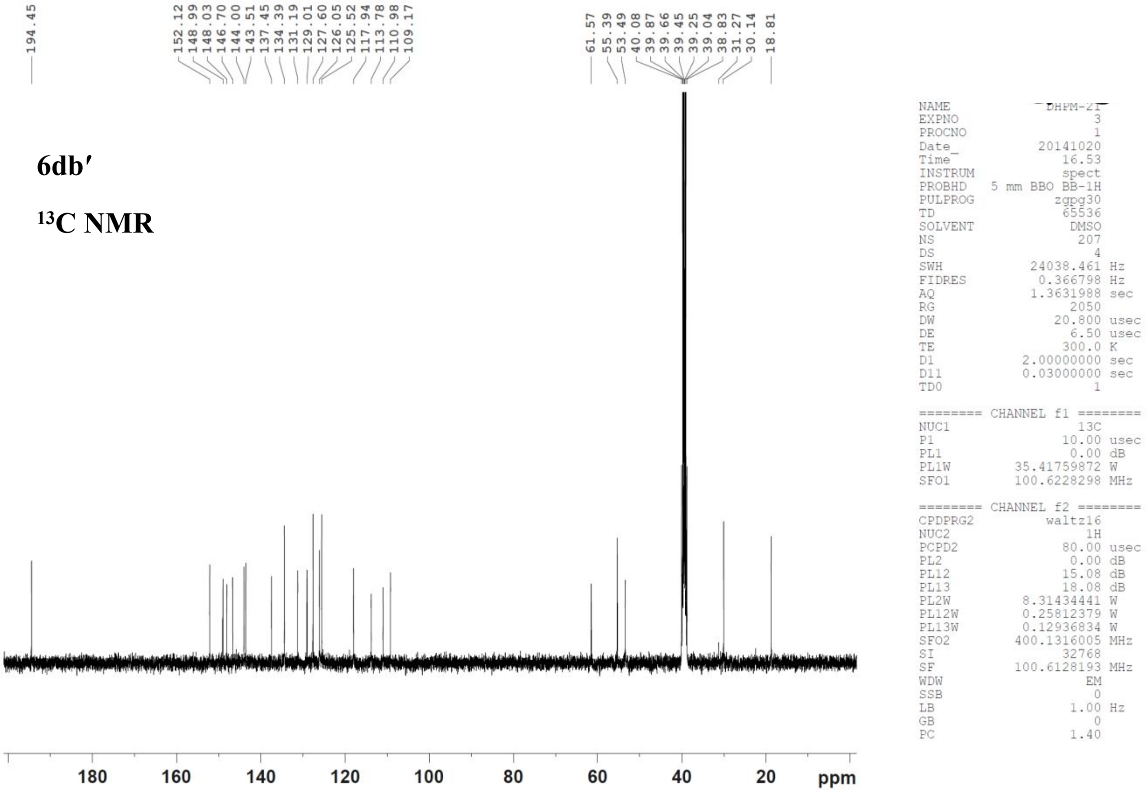

Mass spectra:

Line#:1 R.Time:3.7(Scan#:384)
MassPeaks:128
RawMode:Averaged 1.5-9.4(119-1073) BasePeak:43.000(899)
BG Mode:None Group 1 - Event 1

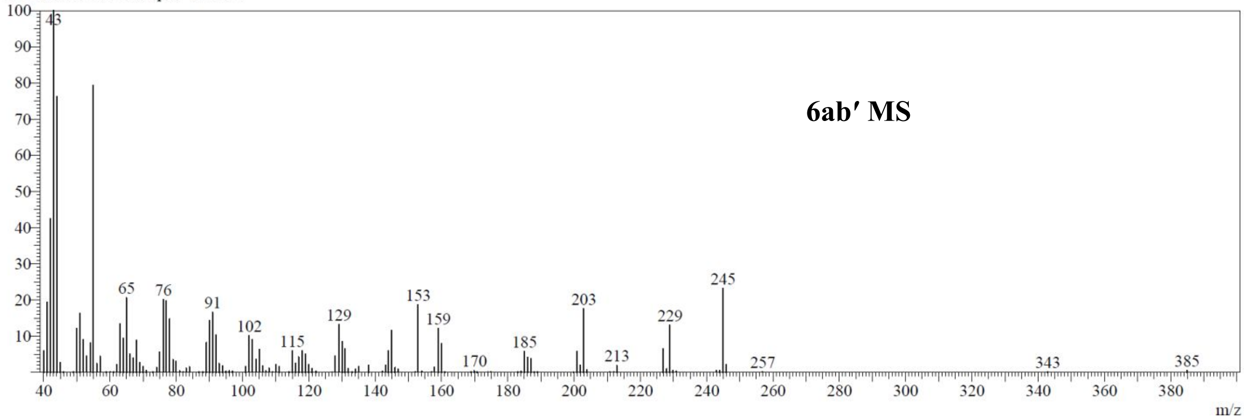

Line#:1 R.Time:4.6(Scan#:488)
MassPeaks:270
RawMode:Averaged 0.6-11.8(15-1357) BasePeak:244.900(5562)
BG Mode:None Group 1 - Event 1

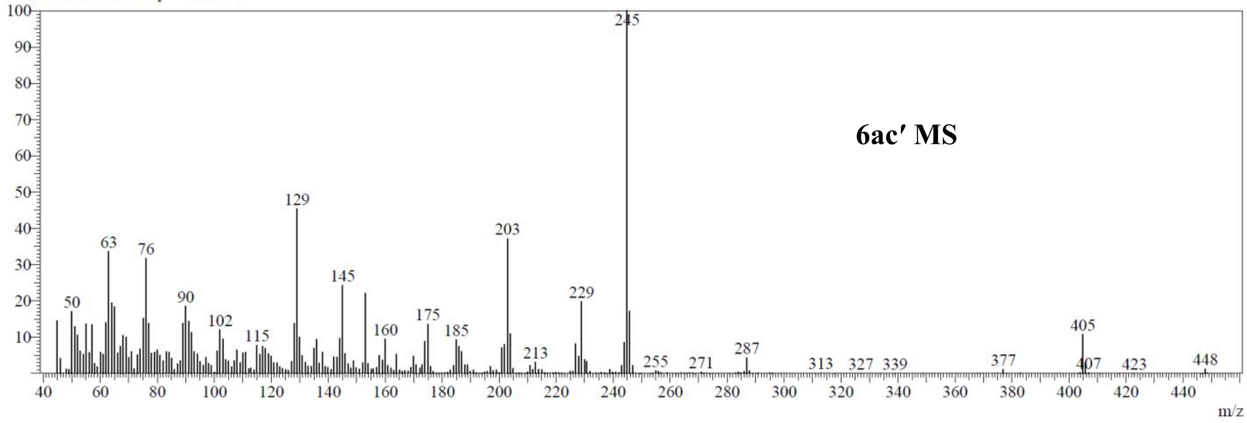

Line#:1 R.Time:3.7(Scan#:385)
MassPeaks:93
RawMode:Averaged 0.7-11.6(22-1335) BasePeak:245.000(692)
BG Mode:None Group 1 - Event 1

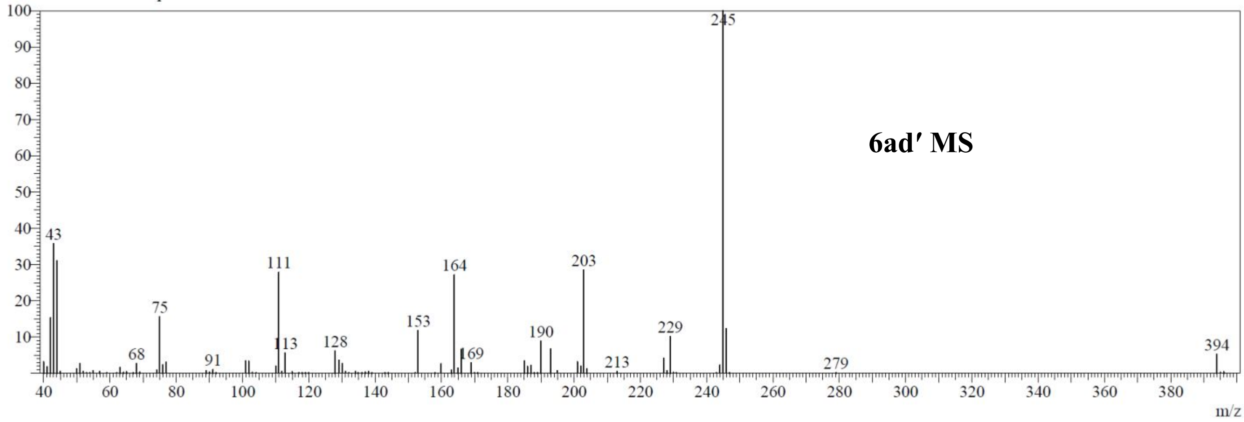

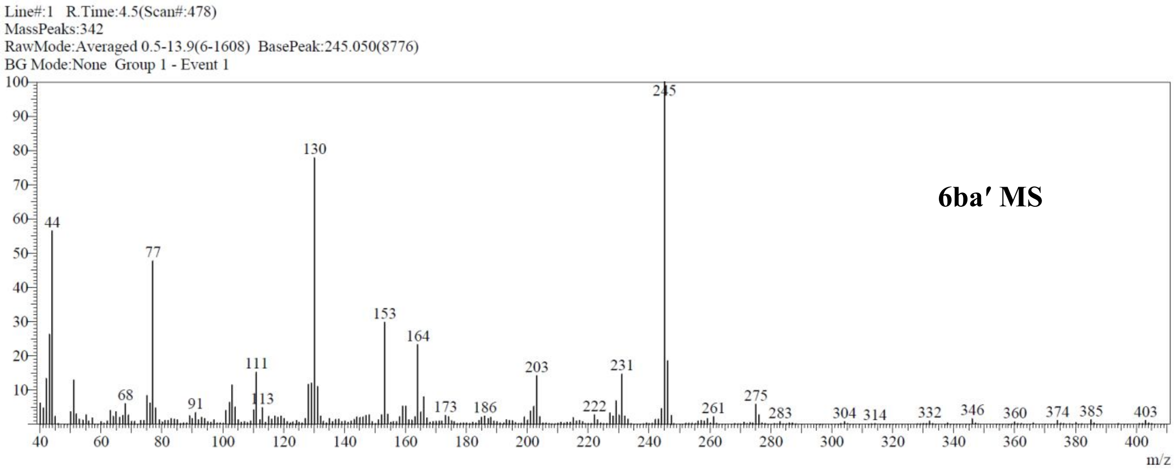
Line#:1 R.Time:4.5(Scan#:478)
MassPeaks:342
RawMode:Averaged 0.5-13.9(6-1608) BasePeak:245.050(8776)
BG Mode:None Group 1 - Event 1
6ba′ MS
m/z

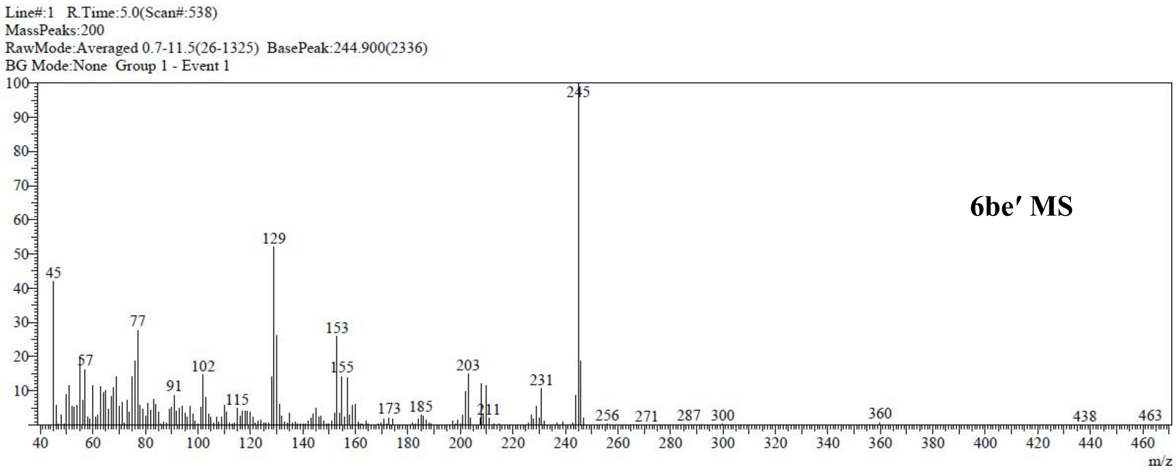
Line#:1 R.Time:5.0(Scan#:538)
MassPeaks:200
RawMode:Averaged 0.7-11.5(26-1325) BasePeak:244.900(2336)
BG Mode:None Group 1 - Event 1
6be′ MS
m/z

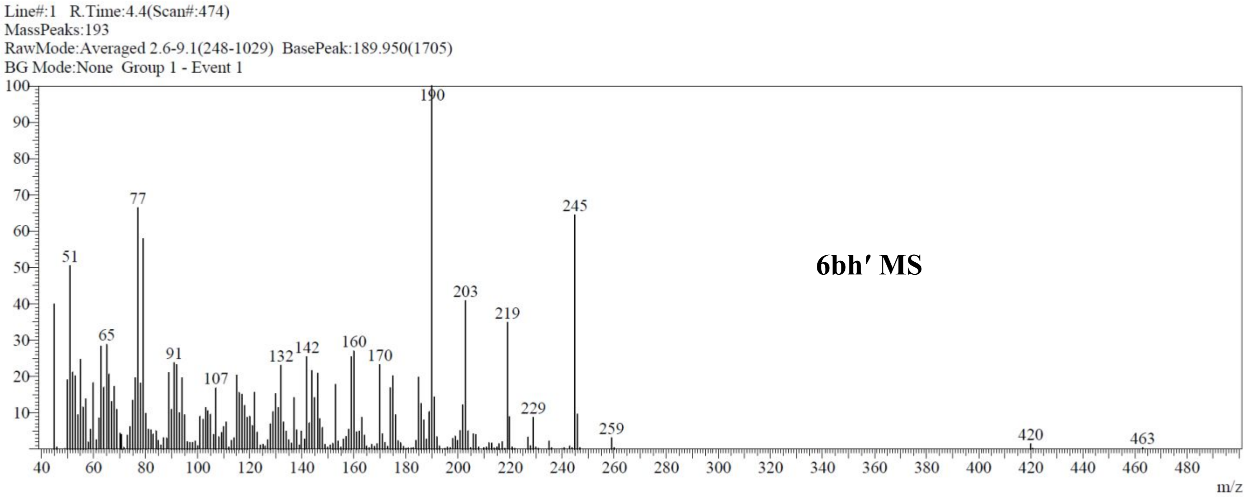
Line#:1 R.Time:4.4(Scan#:474)
MassPeaks:193
RawMode:Averaged 2.6-9.1(248-1029) BasePeak:189.950(1705)
BG Mode:None Group 1 - Event 1
6bh′ MS
m/z

Line#:1 R.Time:6.3(Scan#:694)
MassPeaks:232
RawMode:Averaged 6.0-10.8(663-1236) BasePeak:77.150(3734)
BG Mode:None Group 1 - Event 1

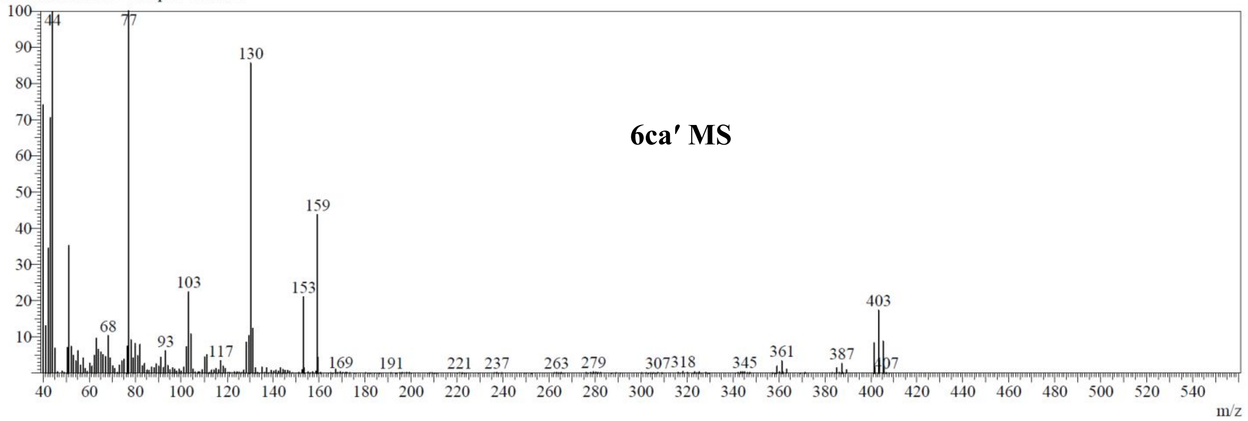

Line#:1 R.Time:4.1(Scan#:435)
MassPeaks:253
RawMode:Averaged 0.5-23.1(1-2718) BasePeak:245.150(679)
BG Mode:None Group 1 - Event 1

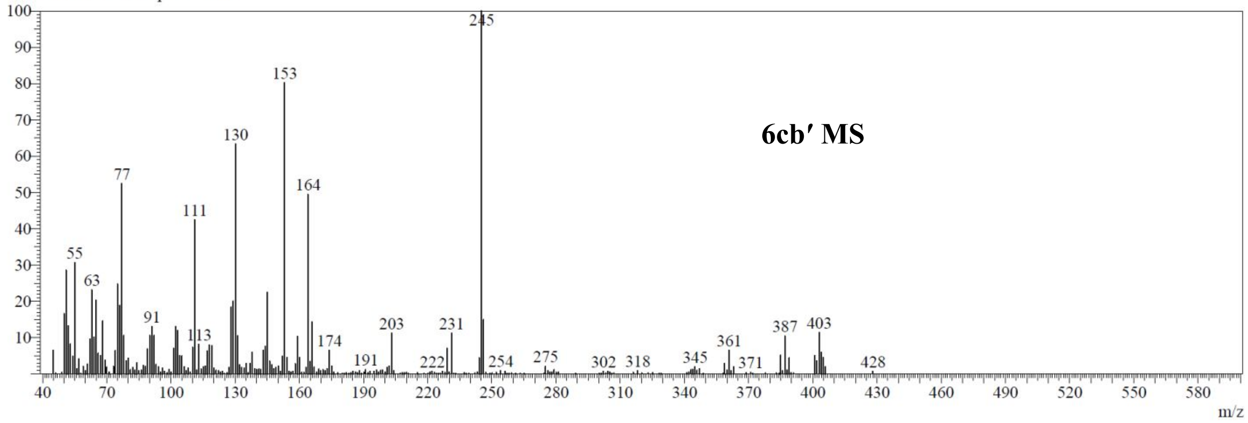

Line#:1 R.Time:4.8(Scan#:518)
MassPeaks:346
RawMode:Averaged 0.8-11.3(32-1291) BasePeak:44.100(20908)
BG Mode:None Group 1 - Event 1

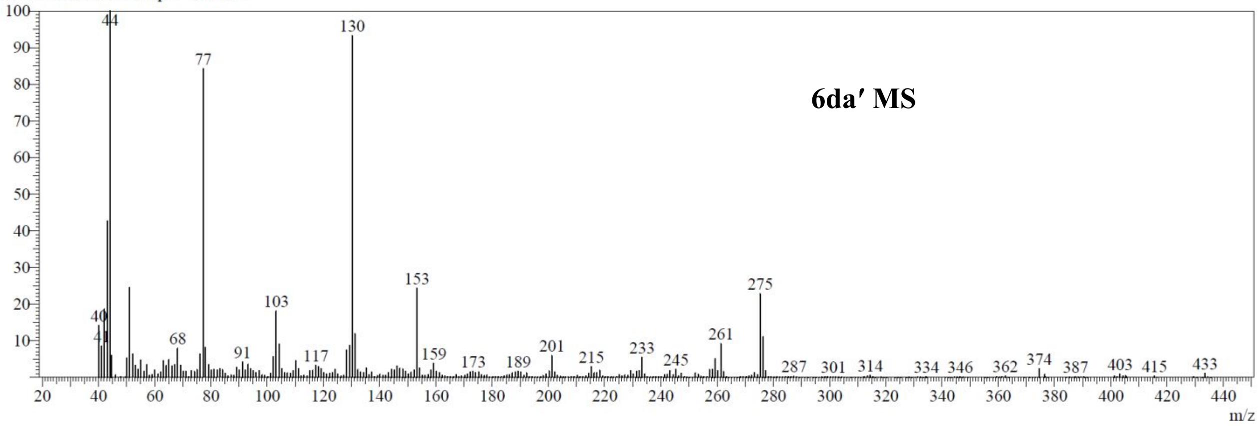

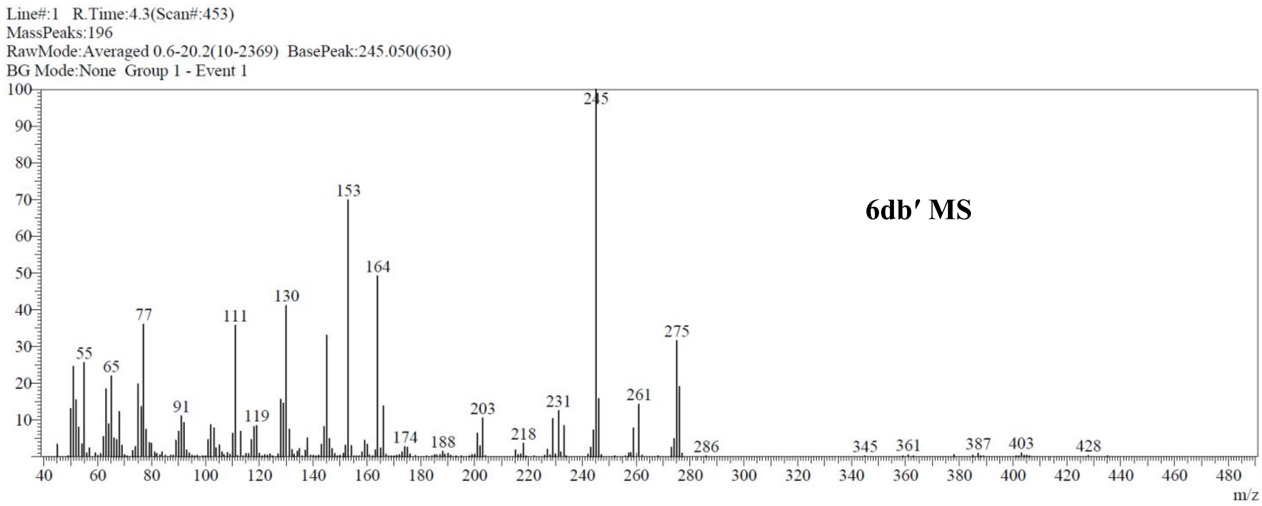
Line#:1 R.Time:4.3(Scan#:453)
MassPeaks:196
RawMode:Averaged 0.6-20.2(10-2369) BasePeak:245.050(630)
BG Mode:None Group 1 - Event 1
6db′ MS
m/z

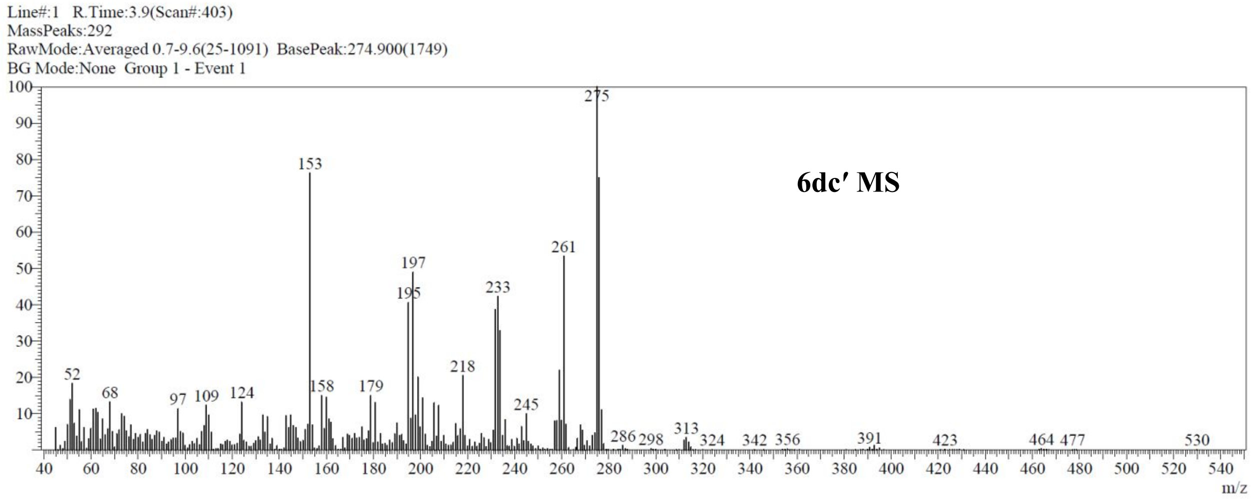
Line#:1 R.Time:3.9(Scan#:403)
MassPeaks:292
RawMode:Averaged 0.7-9.6(25-1091) BasePeak:274.900(1749)
BG Mode:None Group 1 - Event 1
6dc′ MS
m/z

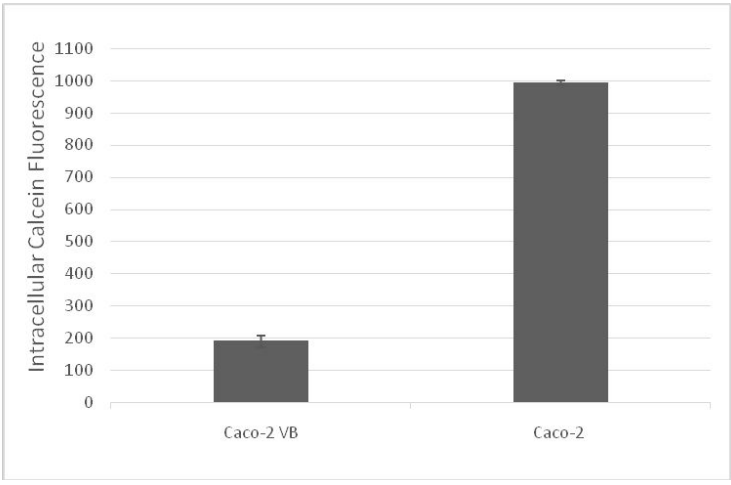

Figure S1 Fluorescent based ABCB1-mediated calcein AM efflux by Caco-2 VB and Caco-2 cells. Intracellular fluorescence intensity of calcein in Caco-2 VB and Caco-2 cells. The mean fluorescent intensities were obtained from Synergy H1 Hybrid Multi-Mode Reader (BioTek). Data represented are mean ± SE of triplicates.

**Compound**	**Cytotoxicity IC_{50} (μM)**
**6aa′**	1.0 ± 0.3
**6ab′**	1.3 ± 0.82
**6ac′**	2.0 ± 0.53
**6ad′**	0.57 ± 0.03
**6ae′**	2 ± 1.2
**6af′**	3.7 ± 0.3
**6ba′**	2.2 ± 0.2
**6bb′**	1.6 ± 0.1
**6bc′**	2.44 ± 0.04
**6bd′**	0.76 ± 0.06
**6be′**	0.8 ± 0.12
**6bf′**	1.50 ± 0.07
**6bh′**	0.6 ± 0.18
**6ca′**	0.9 ± 0.11
**6cb′**	1.22 ± 0.08
**6cc′**	1.25 ± 0.08
**6da′**	2.56 ± 0.04
**6db′**	2.29 ± 0.09
**6dc′**	2.97 ± 0.03
**Carboplatin**	7 ± 2
**Gemcitabine**	4.5 ± 0.98
**Daunorubicin**	14 ± 1.7
**Verapamil**	N.A.
**Cyclosporin A**	N.A.

Table S3: Biological activities of the novel functionalized DHPMs against the Caco-2 cell line

	M-site	H-site	R-site	ATP-site
6aa’ (*R*)	-8.8	-2.4	-6.8	-4.1
6aa’ (*S*)	-6.8	-5.1	-7.2	-5.0
6ab’ (*R*)	-6.5	-3.5	-5.9	-4.2
6ab’ (*S*)	-6.8	-4.9	-6.6	-4.3
6ad’ (*R*)	-8.8	-3.7	-6.5	NA
6ad’ (*S*)	-6.1	-3.0	-6.7	NA
6bd’ (*R*)	-5.3	-3.5	-5.1	NA
6bd’ (*S*)	-6.0	-3.6	-6.0	-4.5
6be’ (*R*)	-8.1	-2.9	-5.8	NA
6be’ (*S*)	-7.4	-3.7	-5.0	NA
6bh’ (*R*)	-6.4	-2.9	-4.4	-4.2
6bh’ (*S*)	-6.8	NA	-4.4	-2.8
6ca’ (*R*)	-9.8	-3.6	-6.2	-4.1
6ca’ (*S*)	-7.4	-3.9	-5.9	-3.9
6cb’ (*R*)	-6.3	-4.3	-6.2	NA
6cb’ (*S*)	-6.7	-2.5	-5.9	NA
6cc’ (*R*)	-8.5	-3.8	-6.1	-3.9
6cc’ (*S*)	-6.7	NA	-5.0	NA

Table S4: Docking scores of functionalized DHPMs docked into M-, H-, R- and ATP- binding sites of hPgp. **Note:** NA = docking not possible due to the size of the site.
